# A novel C5-*O*-methyltransferase for naringenin refines the biosynthetic strategy for polymethoxyflavones

**DOI:** 10.1093/hr/uhag128

**Published:** 2026-04-06

**Authors:** Honglu Hu, Zhenkun Liao, Chenwen Zhou, Bin Liao, Xiaojuan Liu, Chenning Zhao, Dengliang Wang, Lili Liu, Jinping Cao, Yue Wang, Chongde Sun

**Affiliations:** Laboratory of Fruit Quality Biology, The State Agriculture Ministry Laboratory of Horticultural Plant Growth, Development and Quality Improvement, Zhejiang Provincial Key Laboratory of Integrative Biology of Horticultural Plants, Zhejiang University, Hangzhou 310058, China; Laboratory of Fruit Quality Biology, The State Agriculture Ministry Laboratory of Horticultural Plant Growth, Development and Quality Improvement, Zhejiang Provincial Key Laboratory of Integrative Biology of Horticultural Plants, Zhejiang University, Hangzhou 310058, China; Laboratory of Fruit Quality Biology, The State Agriculture Ministry Laboratory of Horticultural Plant Growth, Development and Quality Improvement, Zhejiang Provincial Key Laboratory of Integrative Biology of Horticultural Plants, Zhejiang University, Hangzhou 310058, China; Laboratory of Fruit Quality Biology, The State Agriculture Ministry Laboratory of Horticultural Plant Growth, Development and Quality Improvement, Zhejiang Provincial Key Laboratory of Integrative Biology of Horticultural Plants, Zhejiang University, Hangzhou 310058, China; Laboratory of Fruit Quality Biology, The State Agriculture Ministry Laboratory of Horticultural Plant Growth, Development and Quality Improvement, Zhejiang Provincial Key Laboratory of Integrative Biology of Horticultural Plants, Zhejiang University, Hangzhou 310058, China; Laboratory of Fruit Quality Biology, The State Agriculture Ministry Laboratory of Horticultural Plant Growth, Development and Quality Improvement, Zhejiang Provincial Key Laboratory of Integrative Biology of Horticultural Plants, Zhejiang University, Hangzhou 310058, China; Institute of Fruit Tree Research, Quzhou Academy of Agriculture and Forestry Science, Quzhou 324000, China; Institute of Fruit Tree Research, Quzhou Academy of Agriculture and Forestry Science, Quzhou 324000, China; Laboratory of Fruit Quality Biology, The State Agriculture Ministry Laboratory of Horticultural Plant Growth, Development and Quality Improvement, Zhejiang Provincial Key Laboratory of Integrative Biology of Horticultural Plants, Zhejiang University, Hangzhou 310058, China; Laboratory of Fruit Quality Biology, The State Agriculture Ministry Laboratory of Horticultural Plant Growth, Development and Quality Improvement, Zhejiang Provincial Key Laboratory of Integrative Biology of Horticultural Plants, Zhejiang University, Hangzhou 310058, China; Laboratory of Fruit Quality Biology, The State Agriculture Ministry Laboratory of Horticultural Plant Growth, Development and Quality Improvement, Zhejiang Provincial Key Laboratory of Integrative Biology of Horticultural Plants, Zhejiang University, Hangzhou 310058, China

## Abstract

Polymethoxyflavones (PMFs) are a distinct class of plant flavonoids with significant therapeutic potential, particularly as anticancer agents. This potent bioactivity is largely attributable to *O*-methylation. However, a major bottleneck for the industrial-scale production of PMFs is the lack of suitable precursors and available *O*-methyltransferases (OMTs), which are essential for constructing a viable biosynthetic pathway. Here, we discovered a caffeic acid *O*-methyltransferase (CsOMT5) whose transcriptional dynamics closely mirrored the PMF levels in the flavedo of sweet orange (*Citrus sinensis*). *In vivo* validation further verified that *CsOMT5* indeed promoted PMF accumulation in citrus. And its recombinant protein possessed broad substrate promiscuity for various PMF intermediates. Notably, CsOMT5 exhibited specialized C5-regioselectivity for naringenin, a readily available precursor for *de novo* synthesis of PMFs. Furthermore, critical residues involved in this methylation activity (N14, T18, I120, I256, and G305) were revealed through mutational analysis. This study not only provides deeper insights into the PMF biosynthetic pathway but also opens new avenues for the industrial biosynthesis of PMFs.

## Introduction

The plant kingdom provides a rich reservoir of natural products that are fundamental to human health. Flavonoids, a prominent group of these specialized metabolites, represent a valuable source of natural medicines [1, 2]. Polymethoxyflavones (PMFs), a subclass of flavonoids, have attracted significant attention due to their notable anticancer, anti-inflammatory, metabolic-protective, and neuroprotective properties, establishing them as promising therapeutic candidates [3–10]. The primary source of PMFs is from the flavedo of *Citrus* species (especially *Citrus sinensis* and *C. reticulata*), where they are produced and stored by secretory tissues as part of the defense system [11–13]. At present, PMFs are almost exclusively obtained through plant extraction [14]. However, the naturally limited availability and high extraction costs of PMFs hinder their large-scale production and application. This obstacle has created a pressing need for more sustainable sources, making the complete elucidation of the PMF biosynthetic pathway as a key objective in metabolic engineering and synthetic biology [15–17].

In the flavonoid biosynthetic pathway, diverse classes of flavonoids (e.g. flavanones, flavones, dihydroflavonols, flavonols) are derived from the central intermediate naringenin through various enzymatic modifications, such as hydroxylation, methylation, and glycosylation [1, 18, 19]. The conversion of naringenin to apigenin, catalyzed by flavone synthase (FNS), is widely believed to gate the PMF biosynthesis branch [20]. A previous study also describes a synthetic route for PMFs starting from apigenin [21]. Even so, the availability of abundant precursors in the PMF biosynthetic network suggests that plants leverage metabolic redundancy to foster resilience, rather than relying solely on a single apigenin. Moreover, the reliance on plant-extracted apigenin also impedes the development of a green and cost-effective process. For these reasons, finding a more accessible and economical precursor, such as naringenin, would significantly advance the proposed blueprint for *de novo* synthesis of PMFs.

The potent bioactivities of PMFs are primarily attributed to their multiple methoxy (-OCH_3_) groups, which are catalyzed by *O*-methyltransferases (OMTs). These groups confer low polarity and high lipophilicity, thereby underpinning their bioactivities [22]. *Citrus* species possess a remarkable diversity and abundance of *OMTs*, with sweet orange (*C*. *sinensis*), ‘Mangshan’ mandarin (*C*. *reticulata*), and ‘Clementine’ mandarin (*C*. *reticulata*) containing the highest number [23]. Recent years have seen reporting of many functional citrus *OMTs,* while their application is limited by distinct and constrained substrate preferences and catalytic regioselectivity. For example, CdFOMT5 exhibits low activity toward some monohydroxy flavones in the absence of *in vivo* validation [24]. CrOMT1, CrOMT2, CitOMT2, and CsCCoAOMT1 share a common reliance on flavonoids containing vicinal hydroxyl groups, though their activity on highly methylated flavones is unproven [25–28]. CcOMT1, a homolog of CrOMT2, has been characterized only at the C3 position of natsudaidain [29]. CreOMT4 and CreOMT5 are capable of modifying most major sites in citrus PMFs, but they show poor activity against early-stage PMF intermediates that are typically more accessible [21]. Consequently, a continuing need exists to discover OMTs that are critical for achieving a large-scale biosynthesis of PMFs. This also provides more possibilities for the future collaborative cooperation and industrial application of the identified OMTs.

In this study, we elucidated the spatiotemporal distribution of PMFs in the flavedo and albedo of ‘Bingtangcheng’ sweet orange (*C. sinensis*). By integrating transcriptome data with tissue-specific accumulation patterns of PMFs, we identified candidate *OMTs* responsible for PMF biosynthetic pathway. Subsequently, we characterized one specific gene, *CsOMT5*, and confirmed its vital role in PMF biosynthesis through homologous overexpression and virus-induced gene silencing (VIGS). *In vitro* assays revealed that recombinant CsOMT5 possessed broad substrate flexibility toward various classes of flavonoids. Notably, CsOMT5 catalyzed a key C5-*O*-methylation step on naringenin, a cost-effective and scalable substrate, thereby offering a promising starting point for *de novo* PMF synthesis. Finally, mutational analysis pinpointed the critical residues underpinning this methylation activity, which provides a mechanistic basis for the artificial regulation of PMF biosynthesis.

## Results

### Differential accumulation of PMFs in the flavedo and albedo

**Figure 1:**
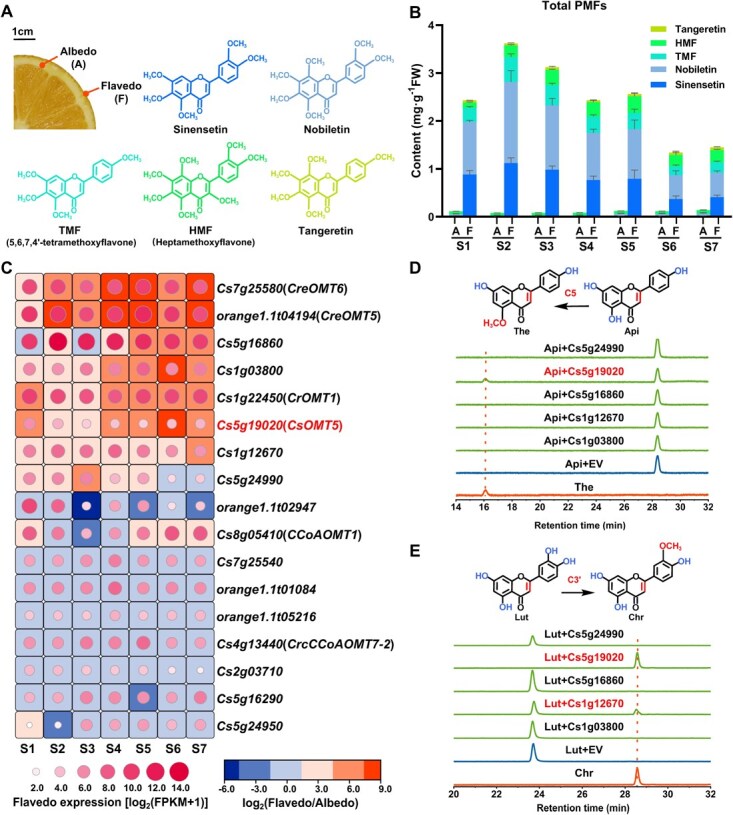
Comparative PMF accumulation and transcriptome analysis of the flavedo and albedo of ‘Bingtangcheng’ across seven developmental stages. (A) Composition and PMF structures in ‘Bingtangcheng’ peel. (B) The total PMF accumulation in the flavedo and albedo of ‘Bingtangcheng’ during maturation. A, albedo; F, flavedo; TMF, 5,6,7,4′-tetramethoxyflavone; HMF, heptamethoxyflavone. (C) Comparative transcriptomes of the flavedo and albedo of ‘Bingtangcheng’ during maturation. FPKM, fragment per kilobase of exon per million fragments mapped. Values are mean ± SD (*n* = 3). (D and E) Incubation of recombinant candidate OMTs with apigenin and luteolin, respectively. EV, empty vector control; Api, apigenin; The, thevetiaflavone; Lut, luteolin; Chr, chrysoeriol. Reaction components are indicated as follows: orange, authentic methylated standards; blue, substrates incubated with EV; green, substrates catalyzed by recombinant candidates. This HPLC analysis was used the flavonoid 50-minute system described in our method.

Citrus peel, comprising the flavedo and albedo (Fig. 1A), is a significant source of PMFs. To elucidate the uncharacterized spatiotemporal distribution between these tissues, we employed HPLC to profile these compounds in ‘Bingtangcheng’ peel across seven developmental stages (S1–S7). Five PMFs—sinensetin, nobiletin, 5,6,7,4′-tetramethoxyflavone (TMF), heptamethoxyflavone (HMF), and tangeretin (Fig. 1A)—were identified throughout fruit development, with sinensetin and nobiletin as the predominant constituents (Fig. 1B). Their accumulation was abundant and dynamic in the flavedo, whereas it was negligible in the albedo. Specifically, the total PMF content in the flavedo initially increased, peaking at approximately 3.6 mg·g^−1^ FW at S2 (60 days after flowering, DAF), before declining thereafter. In the albedo, however, only trace amounts of HMF and nobiletin were detected (0.05 and 0.04 mg·g^−1^ FW, respectively), while the levels of the other three PMFs remained at or below 0.01 mg·g^−1^ FW. These findings reveal a clear preferential accumulation of PMFs in the flavedo. Furthermore, in contrast to the total PMFs, the total flavanones (narirutin, hesperidin, and didymin) exhibited no significant differences in concentration between the flavedo and albedo during maturation (Supplementary Fig. S1).

To further leverage the distinct tissue-specific patterns of PMFs, we performed transcriptome profiling of the flavedo and albedo from ‘Bingtangcheng’ across seven developmental stages. By combining comparative analysis of *OMT* expression profiles with their high abundance in the flavedo, we identified candidate genes potentially involved in the PMF biosynthetic pathway. From these, five previously uncharacterized *OMTs* (*C1g03800*, *Cs1g12670*, *Cs5g16860*, *Cs5g19020*, *Cs5g24990*) were prioritized for subsequent analysis (Fig. 1C).

We successfully cloned these candidates from the cDNA library of ‘Bingtangcheng’ flavedo and purified their proteins by heterologous expression in *Escherichia coli*. In order to identify functional *OMT* genes, the purified proteins were incubated with representative flavones (apigenin and luteolin) *in vitro*. Enzyme activity assays revealed that only Cs1g12670 and Cs5g19020 exhibited detectable activity against these substrates (Fig. 1D and E). In view of the weak activity and restricted substrate range of Cs1g12670, the focus was directed towards Cs5g19020 (designated CsOMT5).

### Identification of *CsOMT5* in PMF biosynthesis

**Figure 2:**
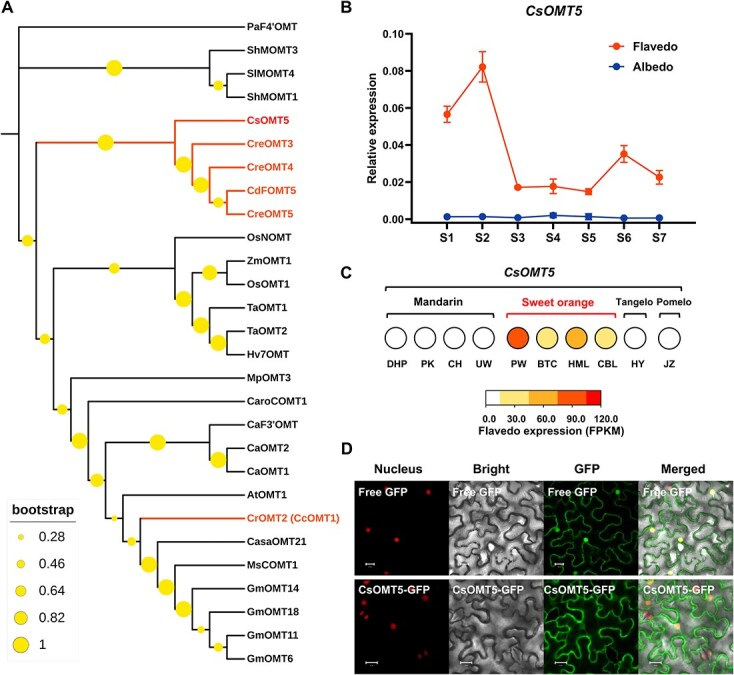
*CsOMT5* is a candidate for PMF biosynthesis. (A) Phylogenetic analysis of CsOMT5 and identified COMTs in other plants. CsOMT5 (Cs5g19020) is labeled in red font and COMTs from citrus are marked in orange. To avoid ambiguity, CrCOMT1 and CsOMT21 were renamed as CaroCOMT1 and CasaOMT21, respectively. See Supplemental Table 1 for COMT sequence details and species origins. (B) The expression pattern of *CsOMT5* in the flavedo and the albedo of ‘Bingtangcheng’ throughout maturation. (C) Expression analysis of *CsOMT5* in flavedo of various citrus cultivars. DHP, ‘Xingyidahongpao’ mandarin (*C. reticulata*); PK, ‘Ponkan’ mandarin (*C. reticulata*); CH, ‘Chachiensis’ mandarin (*C. reticulata*); UW, ‘Ueno wase’ mandarin (*C. unshiu*); PW, ‘Powell’ sweet orange (*C. sinensis*); BTC, ‘Bingtangcheng’ sweet orange (*C. sinensis*); HML, ‘Hamlin’ sweet orange (*C. sinensis*); CBL, ‘Campbell’ sweet orange (*C. sinensis*); HY, ‘Huyou’ tangelo *(C. reticulata* × *C. sinensis*/*C. grandis*); JZ, ‘Jiangxizaoyou’ pomelo (*C. grandis*). (B and C) Data are mean ± SD (*n* = 3). (D) Subcellular localization of CsOMT5 in *N. benthamiana* leaves. Transiently expressed CsOMT5-GFP co-localizes with the nuclear marker mCherry in epidermal cells. Nucleus, mCherry fluorescence; bright, bright-field; GFP, GFP fluorescence; merged, merged channels. Scale bars = 20 μm.

The observed molecular mass of recombinant CsOMT5 was approximately 55 kDa (Supplementary Fig. S2). Protein structural analysis reveals that CsOMT5 contains two conserved domains, Methyltranferase_2 (pfam00891) and Dimerization (pfam08100) (https://smart.embl.de/). The results of molecular mass and conserved domains collectively indicate that CsOMT5 belongs to the COMT family. Next, we constructed a phylogenetic tree with identified COMTs from citrus and other plant species to elucidate the evolutionary relationship of CsOMT5 (Fig. 2A; Supplementary Fig. S3). Phylogenetic analysis placed CsOMT5 in a clade with CreOMT3, CreOMT4, CreOMT5, and CdFOMT5. All of these have been determined to be crucial for PMF biosynthesis and to exhibit multisite *O*-methylation activities for flavonoids [21, 24]. Consequently, we hypothesize that CsOMT5 share a similar catalytic function.

The expression pattern of *CsOMT5* in the flavedo and the albedo of ‘Bingtangcheng’ at seven developmental stages was verified by RT-qPCR (Fig. 2B). *CsOMT5* was specifically expressed in the flavedo, with transcript abundance peaking at S2. Conversely, its levels in the albedo were consistently lower across all stages. The overall expression trend of *CsOMT5* exhibited a mirroring effect on that of PMF accumulation in both tissues, suggesting that *CsOMT5* is a key gene involved in the flavedo-specific biosynthesis of PMFs. In addition, we profiled the expression of *CsOMT5* in the flavedo of diverse citrus species, based on transcriptome data from ten representative cultivars (Fig. 2C). Intriguingly, *CsOMT5* was highly expressed in sweet orange but negligible in mandarin, tangelo, and pomelo, indicating a species-specific expression pattern.

For the subcellular localization of CsOMT5 in plants, we transiently expressed a C-terminal GFP fusion vector (CsOMT5-GFP) in *Nicotiana benthamiana* leaves. A nucleus-located mCherry marker was co-expressed to define nucleus boundaries. As shown in Fig. 2D, CsOMT5-GFP exhibited a diffuse localization pattern throughout the cytoplasm and nucleus, similar to the distribution of free GFP. Dual localization in the cytoplasm and nucleus has been consistently observed for homologous enzymes in citrus and other plant species [28, 30].

### *In vivo* functional validation of *CsOMT5*

**Figure 3:**
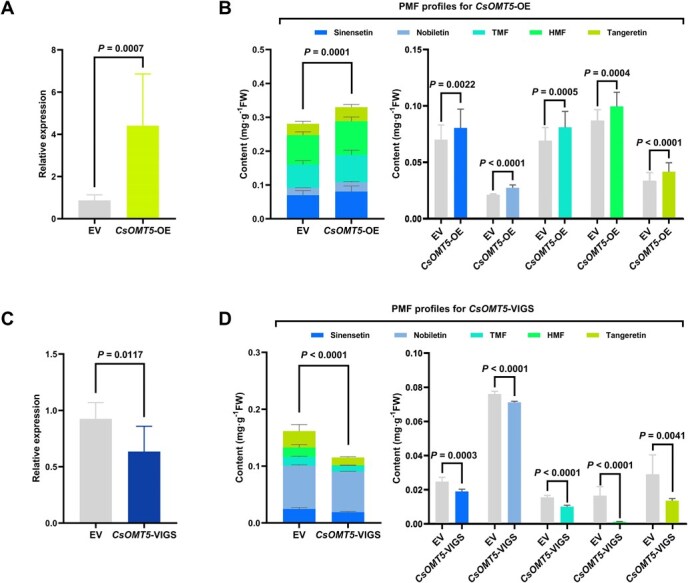
*In vivo* functional characterization of *CsOMT5*. (A) Expression pattern of *CsOMT5* after infiltration with *CsOMT5*-pBI121. (B) PMF profiles from the ‘Bingtangcheng’ peel with overexpression of *CsOMT5*. (C) Relative expression of *CsOMT5* in virus-induced *CsOMT5*-silencing ‘Jincheng’ seedlings. (D) PMF profiles in ‘Jincheng’ seedlings after virus-induced *CsOMT5* silencing. EV, empty vector control. All data are presented as mean ± SD. (A and B) *n* = 10, one-tailed paired *t*-test. (C and D) *n* = 6, one-tailed unpaired *t*-test.

To evaluate the role of *CsOMT5* in enhancing PMF accumulation, we performed homologous overexpression in ‘Bingtangcheng’ peel, where PMFs predominantly accumulate. RT-qPCR analysis confirmed that the relative expression of *CsOMT5* was significantly upregulated approximately 4-fold over the empty vector control (EV; Fig. 3A). Consistent with this result, the total PMF content in the overexpression peels showed a 1.17-fold increase compared to that in the EV (*P* = 0.0001) (Fig. 3B). The contents of specific PMFs (i.e., sinensetin, nobiletin, TMF, HMF, and tangeretin) were found to be elevated by 1.15-, 1.29-, 1.17-, 1.15-, and 1.24-fold, respectively.

Furthermore, to provide negative validation of the functional role of *CsOMT5*, we utilized VIGS in ‘Jincheng’ (*C. sinensis*) seedlings. All six independent VIGS lines showed a significant decrease (approximately 0.63-fold) in the expression of *CsOMT5* (Fig. 3C). This silencing led to a 28.72% reduction in the total PMF content compared with the control plants (*P* < 0.0001) (Fig. 3D), along with a concurrent decline in all individual PMF. These results collectively demonstrate that *CsOMT5* plays a positive role in PMF biosynthesis.

### *In vitro* enzyme assays of recombinant CsOMT5

The enzymatic activity of recombinant CsOMT5 *in vitro* was investigated using *S*-adenosyl-_L_-methionine (SAM) as the methyl donor. The assay was conducted with a diverse range of phenolic substrates, including flavanones, flavones, dihydroflavonols, flavonols, and caffeic acid. Methylated products were identified by comparing their retention times and MS/MS fragmentation patterns with those of authentic standards (Supplementary Fig. S4). For the products lacking commercially available standards, methylation sites were inferred from the behavior of similar substrates, mass spectra, or published literature.

**Figure 4:**
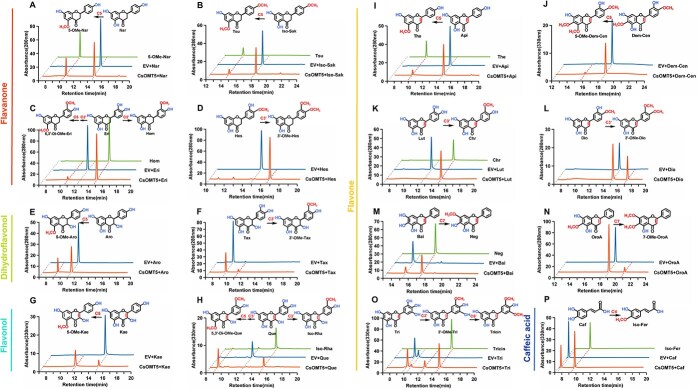
Enzymatic activity of recombinant CsOMT5 via HPLC. Reaction components are indicated as follows: orange, substrates catalyzed by recombinant CsOMT5; blue, substrates incubated with the empty vector control (EV); green, authentic methylated standards. (A–D) Assays with flavanones: Nar, naringenin; 5-OMe-Nar, 5-methylnaringenin; Iso-Sak, isosakuranetin; Tsu, tsugafolin; Eri, eriodictyol; Hom, homoeriodictyol; 5,3′-Di-OMe-Eri, 5,3′-dimethyleriodictyol; Hes, hesperetin; 3′-OMe-Hes, 3′-methylhesperetin. (E and F) Assays with dihydroflavonols: Aro, aromadendrin; 5-OMe-Aro, 5-methylaromadendrin; Tax, taxifolin; 3′-OMe-Tax, 3′-methyltaxifolin. (G and H) Assays with flavonols: Kae, kaempferol; 5-OMe-Kae, 5-methylkaempferol; Que, quercetin; Iso-Rha, isorhamnetin; 5,3′-Di-OMe-Que, 5,3′-dimethylquercetin. (I–O) Assays with flavones: Api, apigenin; The, thevetiaflavone; Dem-Cen, desmethoxycentaureidin; 5-OMe-Dem-Cen, 5-methyldesmethoxycentaureidin; Lut, luteolin; Chr, chrysoeriol; Dio, diosmetin; 3′-OMe-Dio, 3′-methyldiosmetin; Bai, baicalein; Neg, negletein; Oro A, oroxylin A; 7-OMe-Oro A, 7-methyloroxylin A; Tri, tricetin; 3′-OMe-Tri, 3′-methyltricetin. (P) Assays with caffeic acid: Caf, caffeic acid; Iso-Fer, isoferulic acid. The MS/MS spectra of enzymatic products were confirmed by comparison with authentic standards in Supplementary Fig. S4.

The flavanones naringenin, isosakuranetin, eriodictyol, and hesperetin were utilized as substrates. CsOMT5 was observed to possess C5-*O*-methylation activity, as evidenced by the conversion of naringenin and isosakuranetin into 5-methylnaringenin and tsugafolin, respectively (Fig. 4A and B). It is noteworthy that this study provides the first evidence of a plant OMT enzyme that regioselectively methylates naringenin at the C5 position. Additionally, CsOMT5 methylated the C3′ position of eriodictyol (vicinal 3',4′-dihydroxy groups) and hesperetin (isolated 3′-hydroxy groups), yielding homoeriodictyol and 3′-methylhesperetin, respectively (Fig. 4C and D). As the reaction time increased, the conversion of homoeriodictyol to its 5-methyl ether derivative (5,3′-dimethyleriodictyol) was observed (Fig. 4C). This was verified by MS/MS data (Supplementary Fig. S4) and the earlier elution time of the 5-methyl ether derivative compared to the substrate.

The flavones apigenin, luteolin, diosmetin, baicalein, oroxylin A, tricetin, and desmethoxycentaureidin were individually incubated with recombinant CsOMT5. Notably, CsOMT5 demonstrated regioselective C5-*O*-methylation in the conversion of apigenin to thevetiaflavone (Fig. 4I) and desmethoxycentaureidin to its 5-methylated derivative (Fig. 4J), but showed no activity against other apigenin derivatives or gardenin A (Supplementary Fig. S5A, B, C, and  F). Similar to its activity with flavanones (eriodictyol and hesperetin), CsOMT5 methylated the C3′ position of luteolin (vicinal 3,′,4′-dihydroxy groups) and diosmetin (isolated 3′-hydroxy groups) to produce chrysoeriol and 3′-methyldiosmetin, respectively (Fig. 4K and L). However, in contrast to the case with eriodictyol, CsOMT5 could not further methylate chrysoeriol at the C5 position (Fig. 4K; Supplementary Fig. S5E). For substrates such as tricetin, which bears both C3′- and C5′- hydroxy groups, CsOMT5 sequentially methylated it to the C3′-monomethylated product (3′-methyltricetin) and finally the 3′,5′-dimethylated product (tricin) (Fig. 4O). Furthermore, CsOMT5 methylated the C7-hydroxy group of baicalein and oroxylin A, yielding negletein and 7-methyloroxylin A, respectively (Fig. 4M and N).

CsOMT5 also demonstrated methylation activity at the C5 or C3′ position on dihydroflavonols (aromadendrin and taxifolin) and flavonols (kaempferol and quercetin). With aromadendrin and kaempferol as substrates, their 5-methyl ether derivatives (5-methylaromadendrin and 5-methylkaempferol, respectively) were detected and exhibited shorter retention times than their respective substrates (Fig. 4E and G), similar to the cases of 5-methylnaringenin (Fig. 4A). For quercetin, the 5-methyl ether derivative (5,3′-dimethylquercetin) was detected following the accumulation of the initial C3′-monomethylated product (isorhamnetin) (Fig. 4H), sharing characteristics with 5,3′-dimethyleriodictyol (Fig. 4C). However, taxifolin was regiospecifically methylated at the C3′ position to yield 3′-methyltaxifolin (Fig. 4F), as evidenced by MS/MS data (Supplementary Fig. S4) and consistent with the pronounced C3′-regioselectivity of CsOMT5. Additionally, CsOMT5 methylated caffeic acid to form isoferulic acid (Fig. 4P), as expected for a member of the COMT subfamily.

### Substrate preference of recombinant CsOMT5

**Figure 5:**
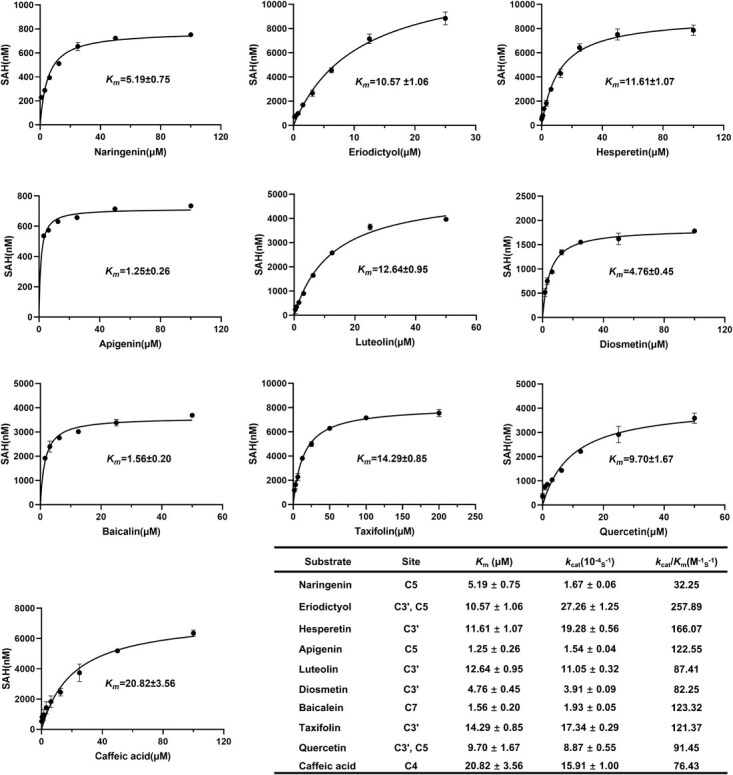
Kinetic parameters of recombinant CsOMT5 with different phenolic substrates. Enzyme activity was determined by measuring the production of *S*-adenosylhomocysteine (SAH; nM). Kinetic parameters were estimated by nonlinear curve fitting of the Michaelis–Menten equation. Data are presented as means ± SD (*n* = 3).

The optimum pH and temperature of recombinant CsOMT5 enzyme were determined to be pH 9.0 and 40°C, with eriodictyol serving as substrate (Supplementary Fig. S6). Under these conditions, enzymatic kinetic assays were performed to characterize the substrate preference of CsOMT5. As shown in Fig. 5, the numerically highest catalytic efficiency of CsOMT5 was observed for eriodictyol (k_cat_/*K*_m_ = 257.89 M^−1^_S_^−1^), followed by hesperetin (k_cat_/*K*_m_ = 166.07 M^−1^_S_^−1^), baicalein (k_cat_/*K*_m_ = 123.32 M^−1^_S_^−1^), apigenin (k_cat_/*K*_m_ = 122.55 M^−1^_S_^−1^), and taxifolin (k_cat_/*K*_m_ = 121.37 M^−1^_S_^−1^). Despite their structural similarity to eriodictyol and hesperetin in possessing vicinal 3′,4′-dihydroxy groups, luteolin (k_cat_/*K*_m_ = 87.41 M^−1^_S_^−1^) and diosmetin (k_cat_/*K*_m_ = 82.25 M^−1^_S_^−1^) were converted by CsOMT5 with lower catalytic efficiency. CsOMT5 exhibited a high affinity toward naringenin (*K*_m_ = 5.19 μM), yet the resulting catalytic efficiency was the lowest among the tested flavonoids (k_cat_/*K*_m_ = 32.25 M^−1^_S_^−1^). In contrast, caffeic acid showed the poorest binding affinity (*K*_m_ = 20.82 μM) and a relatively low catalytic efficiency (k_cat_/*K*_m_ = 76.43 M^−1^_S_^−1^). In summary, CsOMT5 preferentially methylates flavonoids containing a 3′-hydroxyl group, without a strict requirement for a vicinal hydroxyl group. Additionally, CsOMT5 methylates the C5 position of the A-ring in flavonoids with a meta-dihydroxyl group as well at the C7 position.

### Critical residues for flavonoid 5-*O*-methyltransferase activity of CsOMT5

**Figure 6:**
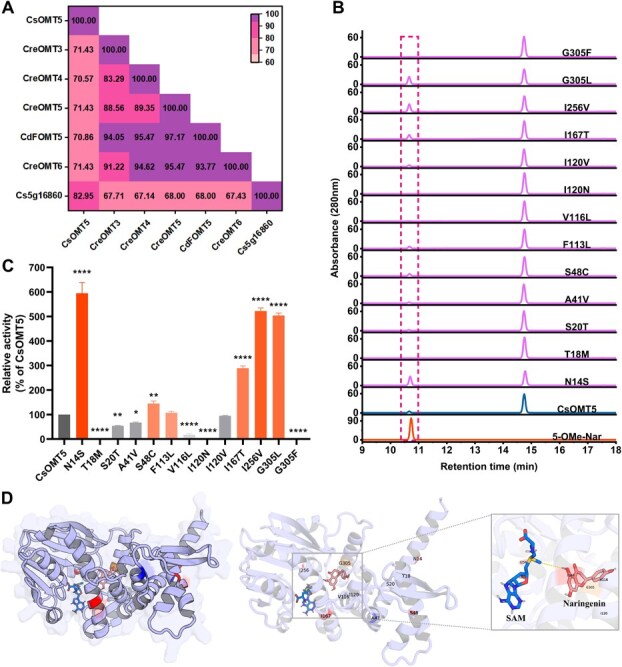
Functional and structural analysis of CsOMT5 mutants. (A) Percentage sequence identity between CsOMT5 and related COMTs. (B) *In vitro* enzyme activity assays of recombinant CsOMT5 mutants with naringenin. Reaction components are indicated as follows: orange, authentic methylated standards (5-OMe-Nar, 5-methylnaringenin); blue, naringenin catalyzed by the wild type (CsOMT5); purple, naringenin incubated with recombinant CsOMT5 mutants. (C) Relative enzymatic activities of CsOMT5 mutants. Data are mean ± SD (*n* = 3). Statistical significance was assessed by one-way ANOVA with Dunnett’s test (^*^P < 0.05, ^**^P < 0.01, ^***^P < 0.001, ^****^P < 0.0001). (D) Molecular model of naringenin and SAM bound to the CsOMT5 substrate pocket. The CsOMT5 structure was modeled using the AlphaFold 3. Docking of naringenin and SAM was performed using AutoDock Vina. Mutated residues are colored according to their functional effect: red, increased activity; blue, abolished activity; orange, G305 (mutations had divergent effects). The yellow dashed line denotes the predicted transfer of the methyl group from SAM to the C5 hydroxyl of naringenin.

Although CsOMT5 shared over 70% sequence identity with CdFOMT5, CreOMT3, CreOMT4, and CreOMT5 (Fig. 6A), these OMTs exhibit distinct substrate preferences and catalytic regioselectivity toward different flavonoid classes [21, 24]. Moreover, CreOMT6 (the previously inferred ancestor of CreOMT3, CreOMT4, and CreOMT5) and Cs5g16860 (high sequence similarity to CsOMT5) are inactive toward flavonoids. To investigate the structural basis underlying this functional divergence, we considered the prevalence of the C5-OCH_3_ modifications in citrus PMFs and the novel C5-*O*-methylation activity of CsOMT5 toward naringenin. Therefore, we selected naringenin as a model substrate and performed multiple sequence alignment of these proteins.

We compared the amino acid sequences of catalytically inactive CreOMT6 and Cs5g16860 with those of functional CreOMT3, CreOMT4, CreOMT5, and CdFOMT5 (Supplementary Fig. S7). A subset of divergent residues in CsOMT5 was selected for site-directed mutagenesis (N14S, T18M, S20T, A41V, S48C, F113L, V116L, I120N, I120V, I167T, I256V, G305L, G305F). The relative activities of the purified wild-type CsOMT5 and mutant proteins were assayed by *in vitro* incubation with naringenin (Supplementary Fig. S8; Fig. 6B). Compared with the wild-type, the N14S, I256V, and G305L mutants exhibited an approximately 5-fold increase in catalytic activity, while the I167T mutant showed a 2.8-fold increase (Fig. 6C). In contrast, the S20T and A41V mutants retained only half of the wild-type activity, and the T18N, I120N, and G305F mutants showed no detectable activity. To interpret these results structurally, molecular docking was performed using a structural model of CsOMT5 predicted by AlphaFold 3 (Fig. 6D). The docking results located V116, I120, I167, I256, and G305 within or near the active site, while N14, T18, S20, A41, and S48 were positioned distal to the catalytic center.

## Discussion

### *CsOMT5* facilitates PMF accumulation in citrus

The accumulation of PMFs is highly specific to the flavedo of citrus fruit throughout its development [11]. Given that the flavedo serves as the primary interface with the external environment, its elevated PMF content contributes to their known defense-related bioactivities, such as protection against pathogens and UV radiation [25, 31, 32]. Based on this tissue-specific accumulation pattern, we compared the transcriptomes of the flavedo and albedo from ‘Bingtangcheng’ sweet orange (Fig. 1C). This comparison revealed a flavedo-specific COMT gene, designated *CsOMT5* (Fig. 2B). The spatiotemporal expression of *CsOMT5* coincided with PMF accumulation (Fig. 1B), suggesting its role in sustaining PMF production during early fruit development and mediating their preferential accumulation in the flavedo. Our previous work also reveals that the transcript levels of *OMTs* associate with the PMF biosynthesis in citrus are upregulated in early development and downregulated during maturation [25, 28]. The observed decline in PMF contents during the later stages of fruit development may be attributable not only to dilution due to fruit expansion but also to competition for flavonoid substrates by diverse modifying enzymes, which may divert the metabolic flux away from the production of PMFs that accumulate as free aglycones [20].

The expression of *CsOMT5* also demonstrated marked species specificity, with high levels detected exclusively in sweet orange and minimal in other species (Fig. 2C). To further investigate this underlying mechanism, we analyzed its homologous genes in representative cultivars (Supplementary Fig. S9A). Phylogenetic analysis revealed that CsOMT5 shares 100% protein sequence identity with CgOMT5 from pomelo, indicating that the coding sequence is not the primary driver of their divergent expression. Subsequent promoter analysis identified a unique flavonoid-related MYB-binding element and two meristem-associated CAT-boxes exclusively in the *CsOMT5* promoter (Supplementary Fig. S9B). These CAT-boxes likely function as transcriptional amplifiers, as this element is indispensable for maintaining promoter potency [33]. Their presence in *CsOMT5* may thus be a key determinant of the robust and flavedo-specific activation observed during fruit development (Fig. 2B). Consistent with this, dual-luciferase assays showed that the *CsOMT5* promoter possesses significantly higher trans-activation activity compared to its homologs (Supplementary Fig. S9C). However, given the moderate activity of the *CgOMT5* promoter in contrast to the negligible gene expression (Fig. 2C), it is likely that multiple regulatory mechanisms, such as divergent *cis*-element landscapes [34], epigenetic constraints [35], and post-transcriptional fine-tuning [36], collectively dictate the specialized accumulation of PMFs in different citrus backgrounds.

Additionally, in light of the near absence of 5-demethylnobiletin in sweet orange versus its predominant accumulation in mandarin [23], the C5-regioselectivity of CsOMT5 toward key PMF intermediates (e.g. naringenin, apigenin and desmethoxycentaureidin) suggests its potential involvement in this differential accumulation. Furthermore, transient overexpression and VIGS of *CsOMT5* significantly increased and decreased the five major PMFs (sinensetin, nobiletin, TMF, HMF, and tangeretin), respectively (Fig. 3B and D). Despite the difficulty in detecting trace early-methylated intermediates due to their high turnover rates [18, 19], these results demonstrate that *CsOMT5* is essential for PMF biosynthesis and positively regulates its production in citrus.

### CsOMT5 is a multifunctional enzyme with a broad preference for flavonoids

CsOMT5 efficiently catalyzes *O*-methylation of various flavonoid substrates at multiple positions (C5, C7, C3′, and C5′) *in vitro*, which aligns with the methylation sites of PMFs confirmed *in vivo* (Figs 1A and 3B and D). Consistent with the behavior of other plant enzymes, this broad substrate promiscuity may represent an evolutionary adaptation [37]. For instance, SbCYP82D1.1 and its duplicate, SbCYP82D2, catalyze the hydroxylation of various flavones, enabling the biosynthesis of baicalein and scutellarein in the roots and aerial parts of *Scutellaria baicalensis* [38]*.*

One of results establishes that CsOMT5 possesses C5-*O*-methylation activity on flavonoids. It preferred flavonoids with a 5,7-dihydroxyl group on the A-ring (e.g. naringenin, apigenin, aromadendrin, and kaempferol) and also methylated analogs with a single pre-existing methyl ether (e.g. isosakuranetin, homoeriodictyol, and isorhamnetin) (Fig. 4). In contrast to these substrates, CsOMT5 was unable to methylate the C5 position of some monomethylated flavonoids, including acacetin, chrysoeriol and 3′-methyltaxifolin, despite their structural similarity to competent substrates (Supplementary Fig. S5C and E; Fig. 4F). These results indicate that the structural features of different flavonoids also influence the C5-regioselectivity of CsOMT5. For downstream flavones in the PMF biosynthesis, CsOMT5 showed a low C5-*O*-methylation activity to desmethoxycentaureidin and was inactivity against gardenin A (Fig. 4J; Supplementary Fig. S5F), while CreOMT4 and CreOMT5 actively catalyze various highly methylated flavones like gardenin B, cirsimaritin, and 5-demethylnobiletin [21]. Those OMTs also differ in their C5-*O*-methylation activity. CreOMT4 and CreOMT5 preferentially methylate the C7 position of flavones with both the C5 and C7 hydroxyl groups, whereas CsOMT5 exhibits a regioselective preference for the C5 position (e.g. apigenin and desmethoxycentaureidin) (Fig. 4I and J).

CsOMT5 also efficiently methylates the C3′ position of flavonoids without dependence on vicinal hydroxyl groups, a difference from our previously reported OMTs (CrOMT1, CrOMT2, and CsCCoAOMT1) [25, 26, 28]. Consequently, CsOMT5 displayed high catalytic ability toward multi-hydroxylated flavonoids (e.g. luteolin and diosmetin) that are poorly methylated by CreOMT3, CreOMT4, and CreOMT5 (Fig. 4K and L) [21]. For tricetin with C3′- and C5′-hydroxyl groups, CsOMT5 methylated both positions to form tricin (3,′,5′-dimethyl ether) (Fig. 4O), a compound exhibiting diverse bioactivities and detected in trace amounts in citrus [39–41]. CsOMT5 also catalyzed C7-methylation of flavonoids (e.g. baicalein and oroxylin A) (Fig. 4M and N). The methylation of CsOMT5 for both the C3′ and C7 positions supports the observation that these are common sites for *O*-methylation in plants [12]. Moreover, the low affinity (*K*_m_ = 20.82 μM) and catalytic efficiency of CsOMT5 for caffeic acid suggest that its primary role in the PMF biosynthesis pathway rather than in the lignin biosynthesis or other phenylpropanoid pathways (Fig. 5) [20, 42, 43].

In brief, CsOMT5 methylates various flavonoids with multiple hydroxyl groups but shows limited activity toward highly methylated flavonoids, suggesting that its function occurs primarily at the upstream stage of the PMF biosynthetic pathway. Thus, with its distinct catalytic profile, CsOMT5 can collaborate with other OMTs to facilitate a more refined PMF pathway [44]. Ultimately, the substrate promiscuity of these OMTs could be further tailored through protein engineering [45], enabling *de novo* biosynthesis of specific PMFs and paving the way for their *in silico* synthesis from scratch.

### Potential methylation of PMFs precedes the formation of the flavone skeleton

CsOMT5 is able to methylate the C5 position of various flavanones, dihydroflavonols, and flavonols (e.g. naringenin, eriodictyol, aromadendrin, and kaempferol). PMFs in citrus typically contain C5-OCH_3_ groups, a feature consistent with the main PMFs identified in ‘Bingtangcheng’ sweet orange in this study (Fig. 1A). Based on those factors, we thus speculate that early-methylated flavonoids could potentially serve as substrates to form PMFs, which challenges the conventional dogma that *O*-methylation of PMFs occurs only after the conversion of flavanones to flavones (e.g. naringenin to apigenin), a reaction catalyzed by FNS [20].

Following our established work [46], the monomethyl ether derivatives of naringenin (5-methylnaringenin, isosakuranetin, and sakuranetin) were separately incubated with CitFNSII-1 and CitFNSII-2. Interestingly, the conversion of isosakuranetin and sakuranetin by CitFNSII-1 and CitFNSII-2 suggests that methylated flavanones can indeed be incorporated into the PMF biosynthetic pathway (Supplementary Fig. S10A and B). Although CitFNSII-1 and CitFNSII-2 appeared inactive toward 5-methylnaringenin in our current assays (Supplementary Fig. S10C), this unconventional route remains strategically significant. Given the vast catalytic plasticity of the plant FNS family, specialized isoforms with such activity might either remain unidentified in nature or be realized through protein engineering [47, 48]. Furthermore, the chemical feasibility of 5-methylnaringenin dehydrogenation, as established by synthetic chemistry [49, 50], highlights this transformation as a viable target for metabolic design. Thus, the regioselective C5-*O*-methylation of naringenin by CsOMT5 provides a foundational scaffold for expanded synthetic biology platforms and *de novo* production of diverse PMFs.

Moreover, the high affinity of CsOMT5 for both naringenin (*K*_m_ = 5.19 μM) and apigenin (*K*_m_ = 1.25 μM) indicates a metabolic flexibility in PMF biosynthesis (Fig. 5), allowing *O*-methylation to occur at early or later stage. Consequently, this positions naringenin at a pivotal branch point in flavonoid metabolism, suggesting that the regulation of naringenin flux is a key target for rational engineering of flavonoid composition, such as flavone glycosides and PMFs [51, 52].

### Critical residues inform the search for novel 5-*O*-methyltransferases

Given that the C5 is an important methylation site in citrus PMFs, it is significant that CsOMT5 regioselectively methylates the C5 position of naringenin, a core precursor in the PMF biosynthesis. Although several plant OMTs can methylate naringenin, such as OsNOMT from rice (*Oryza sativa*) at the C7 position and Pa4′OMT from liverwort (*Plagiochasma appendiculatum*) at the C4′ position [53, 54], no enzyme has been reported to methylate the C5 position.

To investigate C5 catalytic mechanism of CsOMT5 in the PMF biosynthesis, we used naringenin as a model substrate. Mutagenesis analysis revealed that specific residues dramatically altered the catalytic activity of CsOMT5 towards the C5 position of naringenin: the N14S, I167T, I256V, and G305L mutations greatly enhanced C5 methylation efficiency, while the T18M, I120N, and G305F mutations abolished it entirely (Fig. 6C). Notably, several of these influential residues, such as N14 and T18, were located beyond the active site (Fig. 6D). Their impact on catalysis is likely mediated through the perturbation of local chemical properties or protein conformation [55, 56]. DynaMut analysis indicated that the N14S substitution optimized the local hydrogen bond network and increased backbone flexibility (Supplementary Fig. S11A) [57], potentially facilitating a more active enzyme conformation. In contrast, while the T18M enhanced hydrophobic packing, the bulky Methionine side chain likely introduced steric hindrance and disrupted critical polar interactions (Supplementary Fig. S11B). These findings underscore the importance of long-range structural effects, demonstrating that maintaining specific local chemistry and flexibility at distal sites is essential for efficient C5-*O*-methylation.

Previous studies also identify two residues (Asn-17 and Thr-168) in CreOMT4 that influence 5-*O*-methylation activity of gardenin B [21]. Interestingly, while the N17D mutation in CreOMT4 abolishes activity, the equivalent mutation in CsOMT5 (N14S) significantly enhances activity (~5-fold) toward naringenin (Fig. 6C), contrasting with the loss-of-function seen in CreOMT4-N17D. Similarly, the T168A mutation in CreOMT4 increases activity by 1.4-fold, whereas introducing a threonine at the aligned position in CsOMT5 (I167T) yields a more substantial 2.8-fold increase. These results demonstrate that fine-tuning these residues can profoundly alter catalytic efficiency. Briefly, these residues are critical for substrate interaction and C5 regioselectivity, and may serve as functional markers for screening novel OMTs capable of C5 methylation.

**Figure 7:**
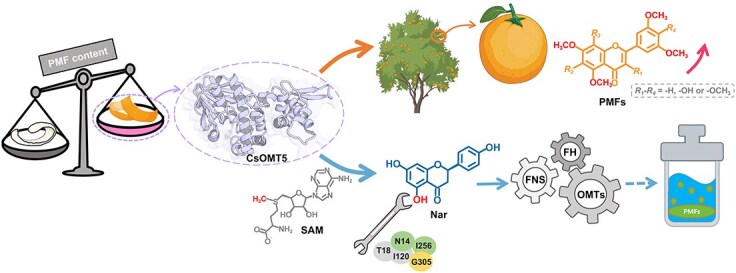
A schematic model of CsOMT5-driven biosynthesis and accumulation of PMFs. SAM, *S*-adenosyl-_L_-methionine; Nar, naringenin; FNS, flavone synthase; FH, flavonoid hydroxylase. The citrus tree and fruit icons were created using BioGDP (https://biogdp.com/) [58].

In conclusion, we characterized a novel gene (*CsOMT5*) in citrus via comparative analysis of PMF contents and OMTs expression levels in the flavedo and albedo of ‘Bingtangcheng’ across different developmental stages. *In vivo* transient overexpression and VIGS experiments further confirmed that *CsOMT5* promotes PMF accumulation. Furthermore, CsOMT5 exhibits broad substrate promiscuity, methylating the C5, C7, C3′, and C5′ positions of highly hydroxylated flavonoids, with a primary role in the upstream steps of PMF biosynthesis. Notably, CsOMT5 is the first enzyme to be reported as methylating the C5 position of naringenin. The mutational analysis demonstrated that residues N14, T18, I120, I256, and G305 are essential for C5 methylation activity. Our findings will contribute to improving the nutrition of citrus fruit and will pave the way for future exploration of the *in silico*-guided *in vitro* synthesis of diverse PMFs for bioactivity research (Fig. 7).

## Materials and methods

### Plant materials and chemicals

Fruits of the ‘Bingtangcheng’ sweet orange (*C. sinensis*) were harvested in Quzhou City, China, at seven developmental stages, each with three biological replicates: S1 (30 days after flowering, DAF), S2 (60 DAF), S3 (80 DAF), S4 (100 DAF), S5 (120 DAF), S6 (150 DAF), and S7 (180 DAF). The flavedo and albedo tissues were dissected and then immediately frozen in liquid nitrogen. Ten citrus cultivars used for expression analysis of *CsOMT5* were collected from Beibei, Chongqing, at the commercial maturity stage. All samples stored at −80°C for future analysis.

Flavonoid standards, including naringenin, sakuranetin, eriodictyol, hesperetin, apigenin, genkwanin, acacetin, luteolin, negletein, oroxylin A, gardenin A, kaempferol, quercetin, and SAM were purchased from Shanghai Yuanye Biotechnology Co., Ltd. (Shanghai, China). 5-Methylnaringenin, tsugafolin, homoeriodictyol, thevetiaflavone, chrysoeriol, aromadendrin, taxifolin, and isoferulic acid were obtained from BioBioPha Co., Ltd. (Kunming, China). Baicalein and isorhamnetin were acquired from Aladdin Scientific Co., Ltd. (Shanghai, China). Tricetin and tricin were sourced from Extrasynthese (Lyon, France) and ChromaDex (Irvine, CA, USA), respectively. Isosakuranetin and diosmetin were procured from CFW Laboratories Inc. (Walnut, CA, USA). Caffeic acid was supplied by Sigma-Aldrich (St. Louis, MO, USA). Desmethoxycentaureidin was acquired by Wuhan ChemFaces Biochemical Co., Ltd. (Wuhan, China). HPLC-grade acetonitrile and methanol were sourced from Tedia Inc. (Fairfield, OH, USA).

### RNA isolation, RNA sequencing, RT-qPCR analysis

Total RNA of samples (‘Bingtangcheng’ developmental stages) was isolated using the RN38-EASYspin Plus Plant RNA Kit (Aidlab Biotechnology Co., Ltd., Beijing, China). All samples from flavedo and albedo at S1 to S7 were used for RNA sequencing (RNA-Seq) (Shenzhen Huada Gene Science and Technology Service Co., Ltd, Shenzhen, China). *OMT* gene expression abundance from RNA-Seq was quantified as Fragments Per Kilobase of transcript per Million mapped reads (FPKM). All sequencing reads were aligned to the *C. sinensis* reference genome (https://www.citrusgenomedb.org/citrus_downloads/Citrus_sinensis/C.sinensis_Hzau_v1.0_genome/).

Reverse transcription quantitative PCR (RT-qPCR) was performed to validate the distinct expression pattern of *CsOMT5* gene. Gene-specific primers were designed using the NCBI Primer-BLAST (https://www.ncbi.nlm.nih.gov/tools/primer-blast/). Gene expression was normalized to Citrus *β*-actin and analyzed using the 2^–ΔΔCt^ method [59]. All the primers are listed in Supplementary Table S2.

### Flavonoid extraction and HPLC analysis

Extraction and detection of flavonoids in citrus were carried out according to our previously study [25], with minor modifications. Samples were ground into a powder in liquid nitrogen. The frozen powder (500 mg) was extracted with 0.5 ml of 80% ethanol for 30 min in an ultrasonic bath. Then, the supernatant was collected via centrifugation at 4000 rpm for 10 min. The samples were extracted twice, and the supernatants were combined (~1 ml). The extract was centrifuged at 13 000 rpm for 30 min before HPLC analysis. A Waters HPLC system (2695 quaternary pump, 2996 diode array detector, Waters Corp., Milford, MA, USA) and a Sunfire C18 ODS column (4.6 × 250 mm, 5 μm; Waters Corp., Milford, MA, USA) were used for flavonoid detection. Acetonitrile (A) and water (B, with 0.1% formic acid) were chosen as the mobile phases with a rate of 1 mL min^−1^. The injection volume was 10 μl and the column temperature was at room temperature. UV spectra were recorded between 280 and 330 nm. The gradient program was as follows: 0 min, 20% A; 5 min, 20% A; 10 min, 27% A; 15 min, 27% A; 25 min, 40% A; 35 min, 60% A; 40 min, 80% A; 42 min, 100% A; 45 min, 20% A; and 50 min, 20% A.

### Phylogenetic analyses and sequence alignment

The amino acid sequence of CsOMT5 was aligned with currently known plant COMT members using the MUSCLE algorithm in MEGA 11.0 software. The phylogenetic tree was constructed via the neighbor-joining method with 1000 bootstrap replicates and beautified and annotated by iTOL (https://itol.embl.de/). UniProt entries of proteins used above are given in Supplementary Table S1. Multiple sequence alignment of CsOMT5 and its homologous counterparts was visualized by ESPript 3.0 (https://espript.ibcp.fr/).

### Gene cloning and recombinant protein purification

Candidate *OMT* genes were isolated based on the *C. sinensis* reference genome (v1.0). The full coding sequence (CDS) without a stop codon was amplified from the flavedo cDNA of ‘Bingtangcheng’ and then inserted into the pET32a (+) vector. After confirming sequences, the recombinant plasmids were transferred into *E. coli* strain BL21 (DE3) pLysS (Shanghai Weidi Biotechnology Co., Ltd., Shanghai, China). The above primers are described in Supplementary Table S2. Protein expression was performed using our established protocol [25]. The fusion proteins were purified using His-Tagged Protein Purification Kit (Reduction-Resistant Chelating Type, P2226, Beyotime Biotechnology, Shanghai, China), then replaced with a Tris–HCl storage buffer (50 mM, pH 9.0, 10% glycerol and 2 mM DTT) via a PD-10 desalting column (GE Healthcare, Uppsala, Sweden), before being stored at −80°C. Protein concentration was measured with Modified BCA Protein Assay Kit (Sangon Biotech, Shanghai, China).

### Transient overexpression in citrus fruit peel

The full-length CDS of *CsOMT5* without a stop codon was constructed onto the pBI121 vector and then transformed into *Agrobacterium tumefaciens* strain EHA105 (Shanghai Weidi Biotechnology Co., Ltd., Shanghai, China), following a previous method [60]. ‘Bingtangcheng’ fruits (S5) were selected based on their uniform size and color and healthy. Specifically, each fruit was treated as one biological replicate, with a total of 10 replicates. The suspensions carrying the target gene *CsOMT5* and the EV were injected into opposite sides of the equatorial plane of each fruit. After infiltration, the fruits were kept in the dark for 12 h and then under a 16 h light/8 h dark photoperiod for 5 d. The peels of injection regions were sampled for analysis of the PMF contents and gene expression by HPLC and RT-qPCR, respectively. Primers for *CsOMT5*-pBI121 and RT-qPCR are listed in Supplementary Table S2.

### TRV-mediated VIGS in citrus seedlings

The TRV-mediated VIGS was based on our previous study [60]. ‘Jincheng’ sweet orange (*C. sinensis*) was chosen for this study due to its abundant seeds and well-established genetic transformation system. A 241-bp CDS of the *CsOMT5* CDS was amplified by PCR, introduced into the TRV2 vector and then transformed into *A. tumefaciens* strain EHA105. The *A. tumefaciens* cells containing the TRV1 and TRV2 recombinant plasmids were cultured in LB broth to OD_600_ = 1.0. Next, the cultures were centrifuged and resuspended in infiltration buffer (10 mM MES, 10 mM MgCl2, 200 μM acetosyringone, pH = 5.6). The cell suspensions of TRV1 and TRV2 were subsequently mixed in 1:1 volume ratio and infiltrated into germinating ‘Jincheng’ seedlings by vacuum method (−100 kPa, 1 min). After infiltration, the seedlings were grown in the dark for 3 d and then moved to a growth chamber for 1 month. The measurement of PMF contents and gene expression was as mentioned above. The relevant primers are provided in Supplementary Table S2.

### Subcellular localization

The full-length CDS of *CsOMT5* without a stop codon was inserted into the *35S*-eGFP vector and then transferred into *A. tumefaciens* strain GV3101 (Shanghai Weidi Biotechnology Co., Ltd., Shanghai, China). The primers are listed in Supplementary Table S2. The transformants carrying positive vectors and the EV were cultured in liquid LB broth and resuspended in MES-NaOH buffer (10 mM MES, 10 mM MgCl_2_, 150 μM acetosyringone, pH = 5.6) to OD_600_ = 0.7. The infection solution was injected into 4-week-old *N. benthamiana* leaves. After 2 d, the samples were examined with a confocal laser scanning microscope (Zeiss LSM 710 NLO, Oberkochen, Germany).

### Enzyme assays and kinetics

Enzymatic assays were performed in a volume of 100 μl, which included Tris–HCl buffer (50 mM, pH 9.0), 1 mM SAM, 100 μM substrate and 10 μl of purified protein. The reactions were incubated at 40°C for 2 h, then stopped by the addition 100 μl methanol. The mixture was centrifuged at 13 000 rpm for 30 min, and the supernatants were analyzed using an Agilent 1290 ultra-HPLC (Agilent Technologies, Santa Clara, CA, USA), equipped with an Inertsil C18 ODS column (4.6 × 250 mm, 5 μm; GL Sciences Inc., Tokyo, Japan). The mobile phases were water (A, with 0.1% formic acid) and acetonitrile (B) at a flow rate of 1 ml min^−1^. The gradient program was as follows: 0 min, 20% B; 2 min, 20% B; 4 min, 30% B; 12 min, 50% B; 19 min, 80%; 21 min, 100% B; 22 min, 100% B; 23 min, 20% B; 26 min, 20% B. Enzymatic products were detected at 280 and 330 nm with a 10-μl injection volume, and were confirmed via liquid chromatography-tandem mass spectrometry (LC–MS/MS) using an AB Sciex TripleTOF 5600+ System (AB Sciex, Framingham, MA, USA), as previously described [46].

The optimum reaction conditions were performed with eriodictyol. The optimum temperature for CsOMT5 was tested at ranging from 25 to 65°C in Tris–HCl buffer (50 mM, pH 9.0). The effect of pH was estimated between pH 5.5 and 10.0 in three different buffers, including potassium phosphate buffer (100 mM, pH 5.5–8.0), Tris–HCl buffer (50 mM, pH 7.0–9.5) and Na_2_CO_3_ − NaHCO_3_ buffer (100 mM, pH 8.5–10.0). Kinetic analysis of the CsOMT5 protein was measured via MTase-Glo™ Methyltransferase Assay Kit (Promega, Madison, WI, USA). The reaction components contained 3 μg of purified protein, 250 μM SAM, various concentrations of flavonoid substrates (0–400 μM) and Tris–HCl buffer (50 mM, pH 8.0), with a volume of 20 μl. Kinetic parameters were estimated by fitting the data to the Michaelis–Menten equation using nonlinear regression in GraphPad Prism 8.0 software.

### Molecular docking and site-directed mutagenesis

The protein structure of CsOMT5 protein was predicted using the AlphaFold 3 AI model via AlphaFold Server (https://alphafoldserver.com/). PlayMolecule (https://open.playmolecule.org/) was used to predict the active pocket of protein. The 3D structures of the naringenin and SAM were obtained from PubChem (https://pubchem.ncbi.nlm.nih.gov/). Naringenin and SAM were docked into the active site using AutoDock Vina 1.5.7 software. The model was viewed and rendered using PyMol 3.1.3.1. Site-directed mutagenesis was performed on CsOMT5 using Mut Express II Fast Mutagenesis Kit V2 (Vazyme, Nanjing, China), following the manufacturer’s instructions. Primes of mutation sites can be found in Supplementary Table S2. The enzyme activity analysis of the mutant recombinant proteins was the same as that of CsOMT5.

### Statistical analysis

Structural formulas were produced using KingDraw 4.0 software. Graphics were generated using GraphPad Prism 8.0 and Origin 2024 Learning Edition. All experiments were repeated at least three times and data were presented as means ± SD. Statistical significance was calculated using one-way ANOVA.

## Supplementary Material

Web_Material_uhag128

## Data Availability

The raw transcriptome data in this study are available at GenBank under project PRJNA924350 and PRJNA923786 [46].
