# Supplementary material for: A novel C5-O-methyltransferase for naringenin refines the biosynthetic strategy for polymethoxyflavones
Source: Hortic Res. 2026 Apr 6;13(8):uhag128. doi: 10.1093/hr/uhag128 (PMC13411268; doi:10.1093/hr/uhag128)
Supplement: Web_Material_uhag128 [file web_material_uhag128.zip › Supplementary Figures.pdf]

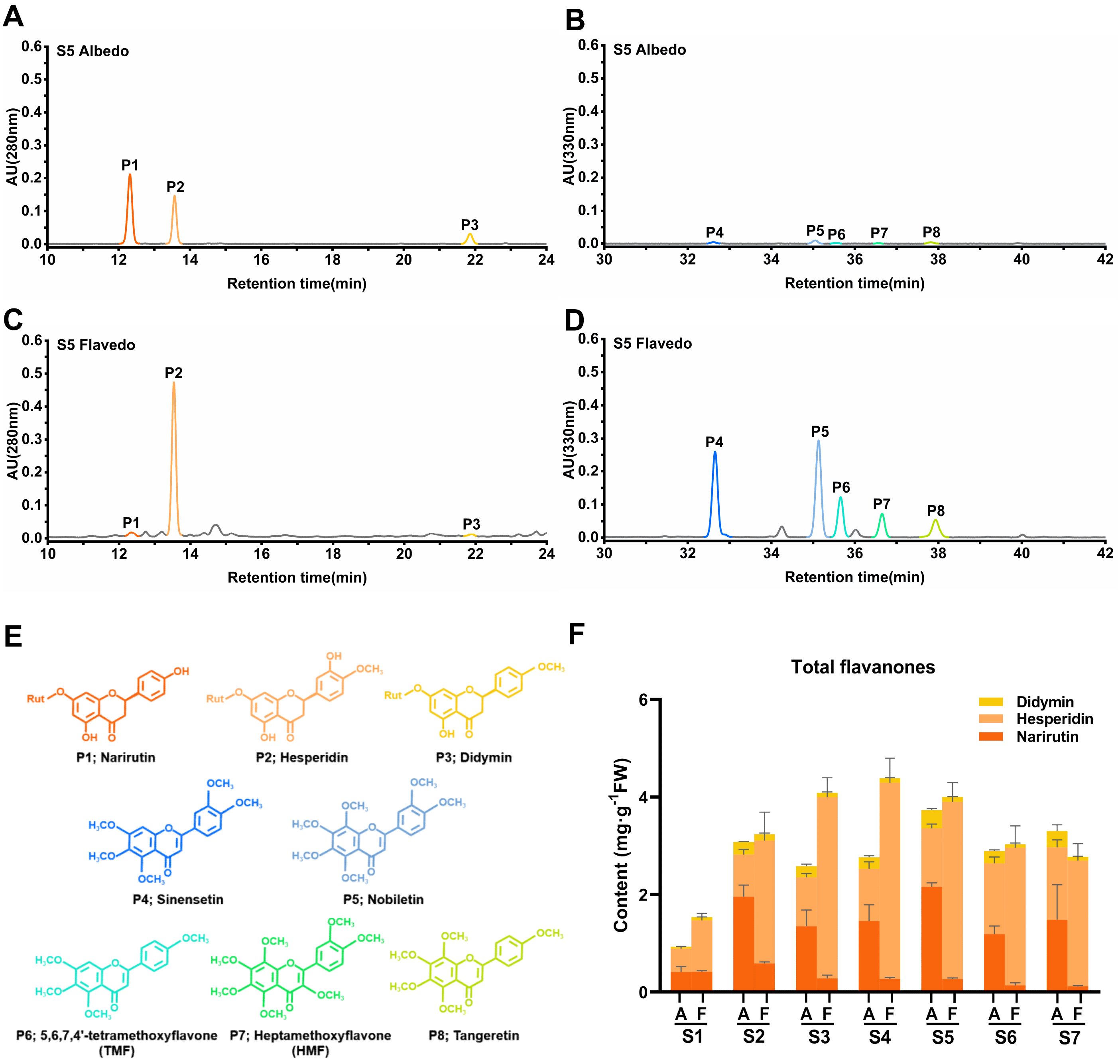

Supplemental Figure 1. Flavonoids in the flavedo and albedo of ‘Bingtangcheng’.

(A-B) Representative HPLC chromatogram showing the flavanones (280 nm) and PMFs (330 nm) in the albedo of ‘Bingtangcheng’ at stage S5. **(C-D)** Representative HPLC chromatogram showing the flavanones (280 nm) and PMFs (330 nm) in the flavedo of 'Bingtangcheng' at stage S5. **(E)** Structural formulae of flavanones (P1-P3) and PMFs (P4-P8). **(F)** The total flavanone accumulation in the flavedo and albedo of ‘Bingtangcheng’ from seven development stages. A, albedo; F, flavedo.

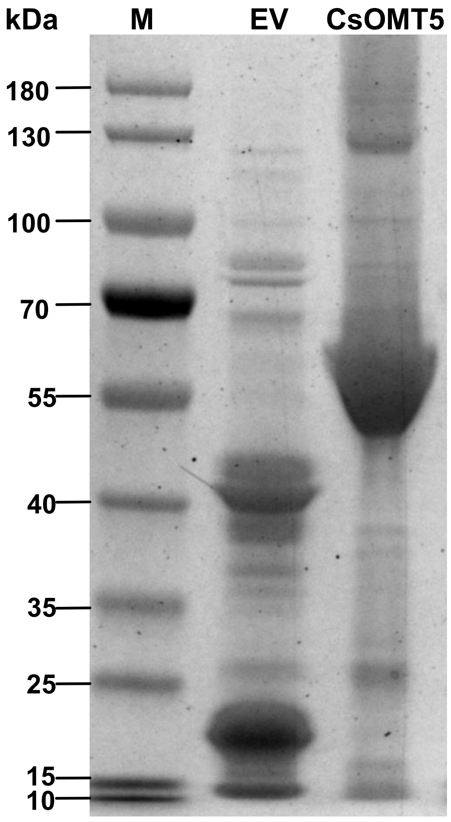

Supplemental Figure 2. SDS-PAGE analysis of recombinant CsOMT5 in *E. coli*.

M, protein marker; EV, empty vector pET32a.

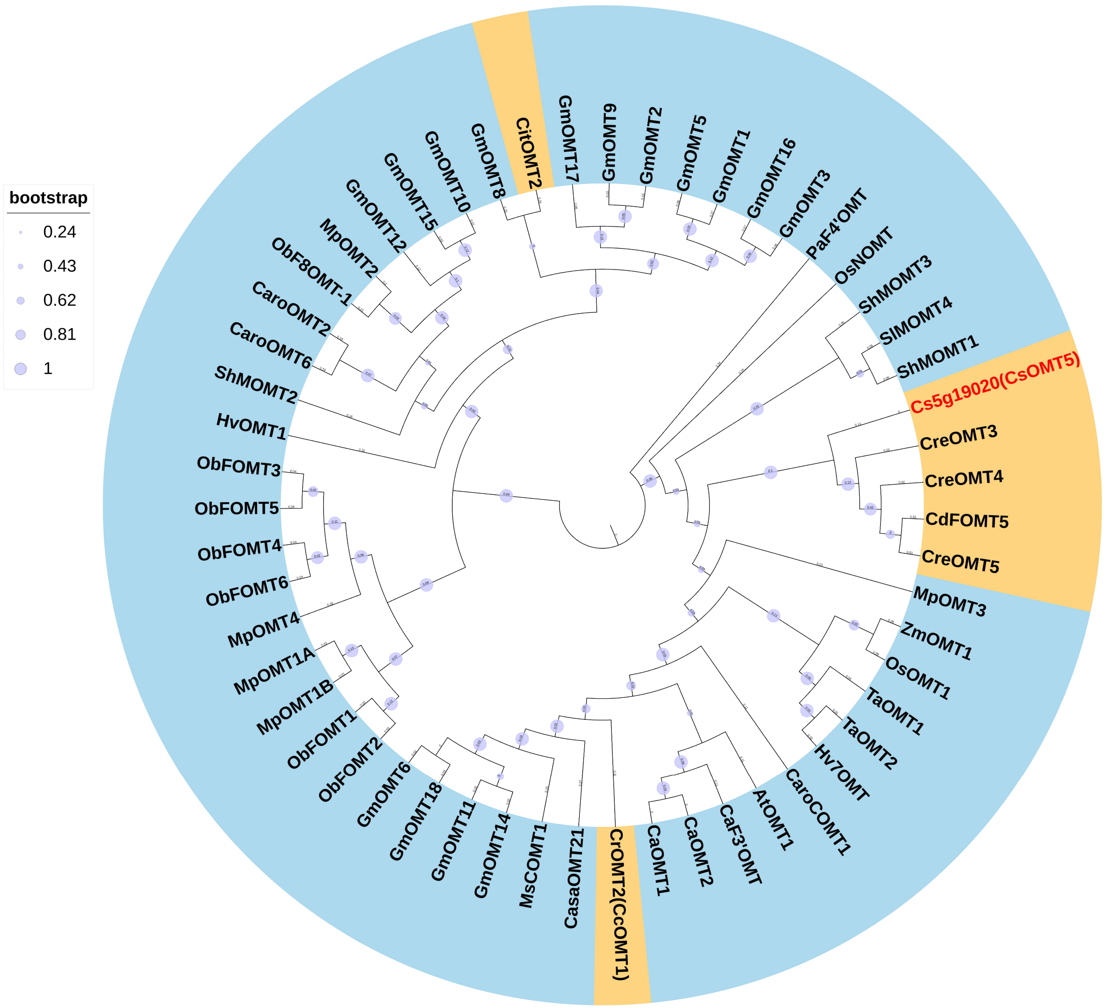

Supplemental Figure 3. Phylogenetic analysis of CsOMT5 and identified COMTs in other plants. COMTs from citrus are highlighted on an orange background, whereas those from other plants are shown on a blue background. CsOMT5(Cs5g19020) in this study is labeled in red font. To avoid ambiguity, CrCOMT1, CrOMT2, CrOMT6, and CsOMT21 were renamed as CaroCOMT1, CaroOMT2, CaroOMT6 and CasaOMT21, respectively. See Supplemental Table 1 for COMT sequence details and species origins.

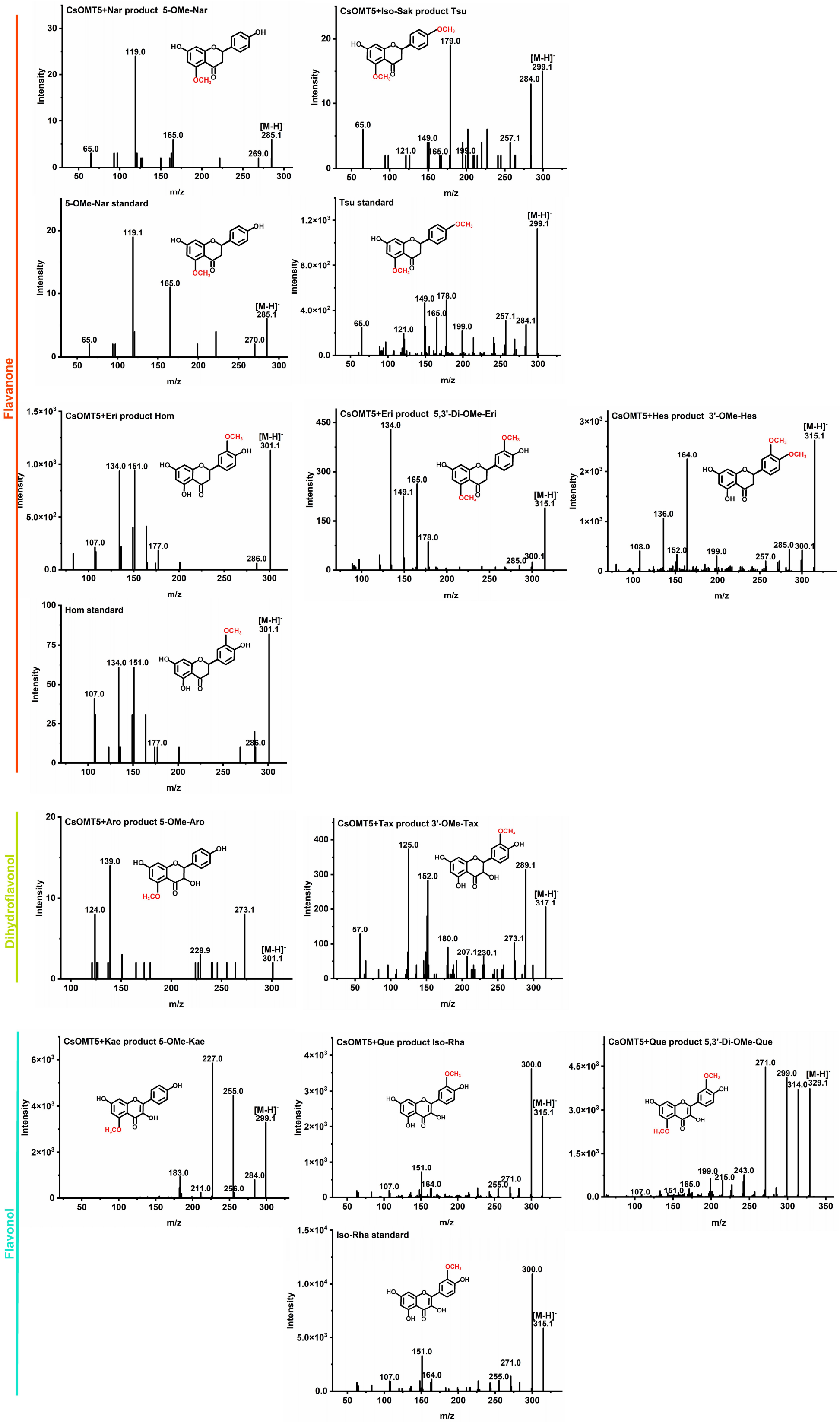

Flavanone
Dihydroflavonol
Flavonol
CsOMT5+Nar product 5-OMe-Nar
CsOMT5+Iso-Sak product Tsu
5-OMe-Nar standard
Tsu standard
CsOMT5+Eri product Hom
CsOMT5+Eri product 5,3'-Di-OMe-Eri
CsOMT5+Hes product 3'-OMe-Hes
Hom standard
CsOMT5+Aro product 5-OMe-Aro
CsOMT5+Tax product 3'-OMe-Tax
CsOMT5+Kae product 5-OMe-Kae
CsOMT5+Que product Iso-Rha
CsOMT5+Que product 5,3'-Di-OMe-Que
Iso-Rha standard
Intensity
m/z
[M-H]-

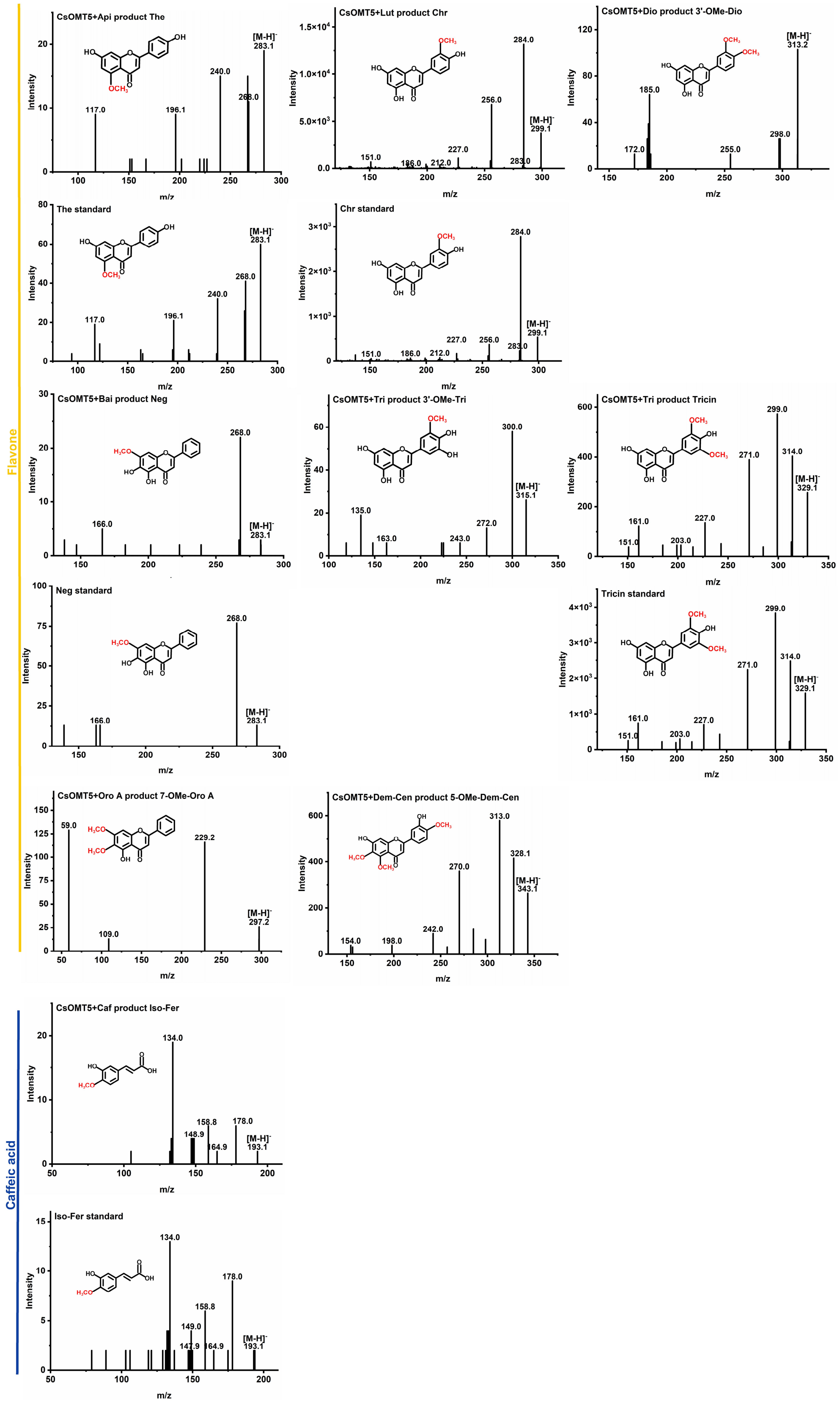

Supplemental Figure 4. MS/MS spectra of methylated products generated by CsOMT5 *in vitro* and corresponding authentic standards.

All spectra were acquired in negative ion mode.

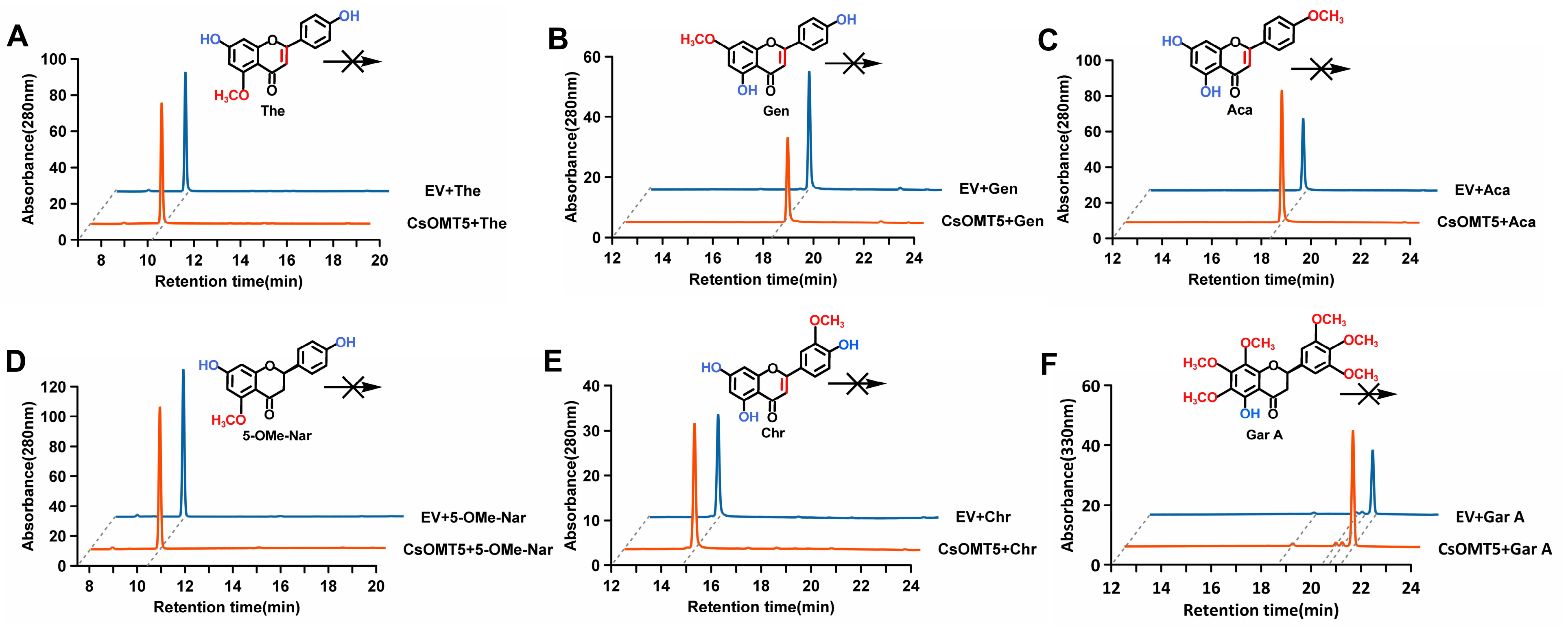

Supplemental Figure 5. ***In vitro*** **enzymatic activity assay between CsOMT5 and its non-methylatable substrate.**

Reaction components are indicated as follows: orange, substrates catalyzed by recombinant CsOMT5; blue, substrates incubated with the empty vector (EV). **(A-C)** Assays with derivatives of apigenin: The, thevetiaflavone; Gen, genkwanin; Aca, acacetin. **(D)** Assays with 5-OMe-Nar (5-methylnaringenin). **(E)** Assays with Chr (chrysoeriol). **(F)** Assays with Gar A (Gardenin A).

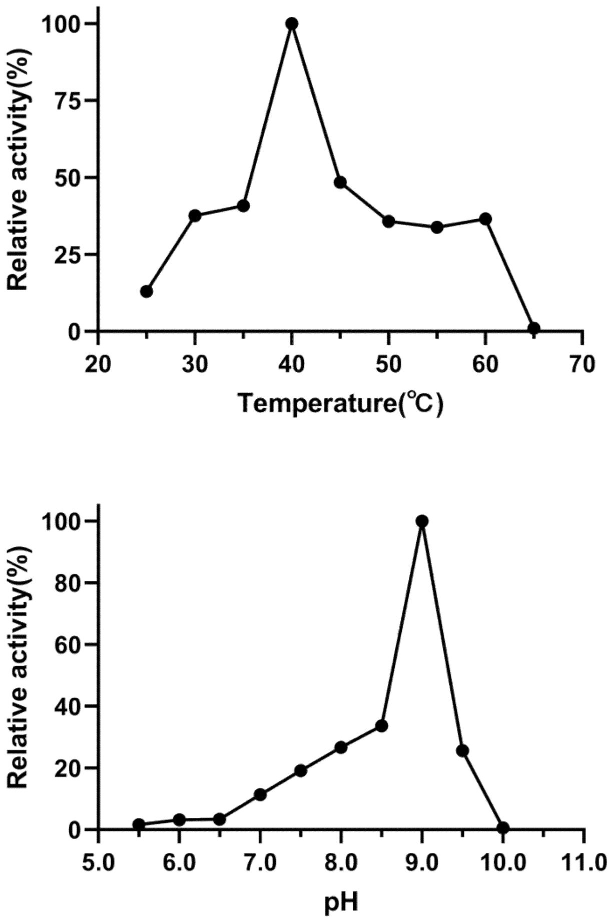

Supplemental Figure 6. Temperature and pH optima of recombinant CsOMT5.

Relative activity (mean ± SD, *n*=3) was based on the HPLC peak area of homoeriodictyol produced from eriodictyol.

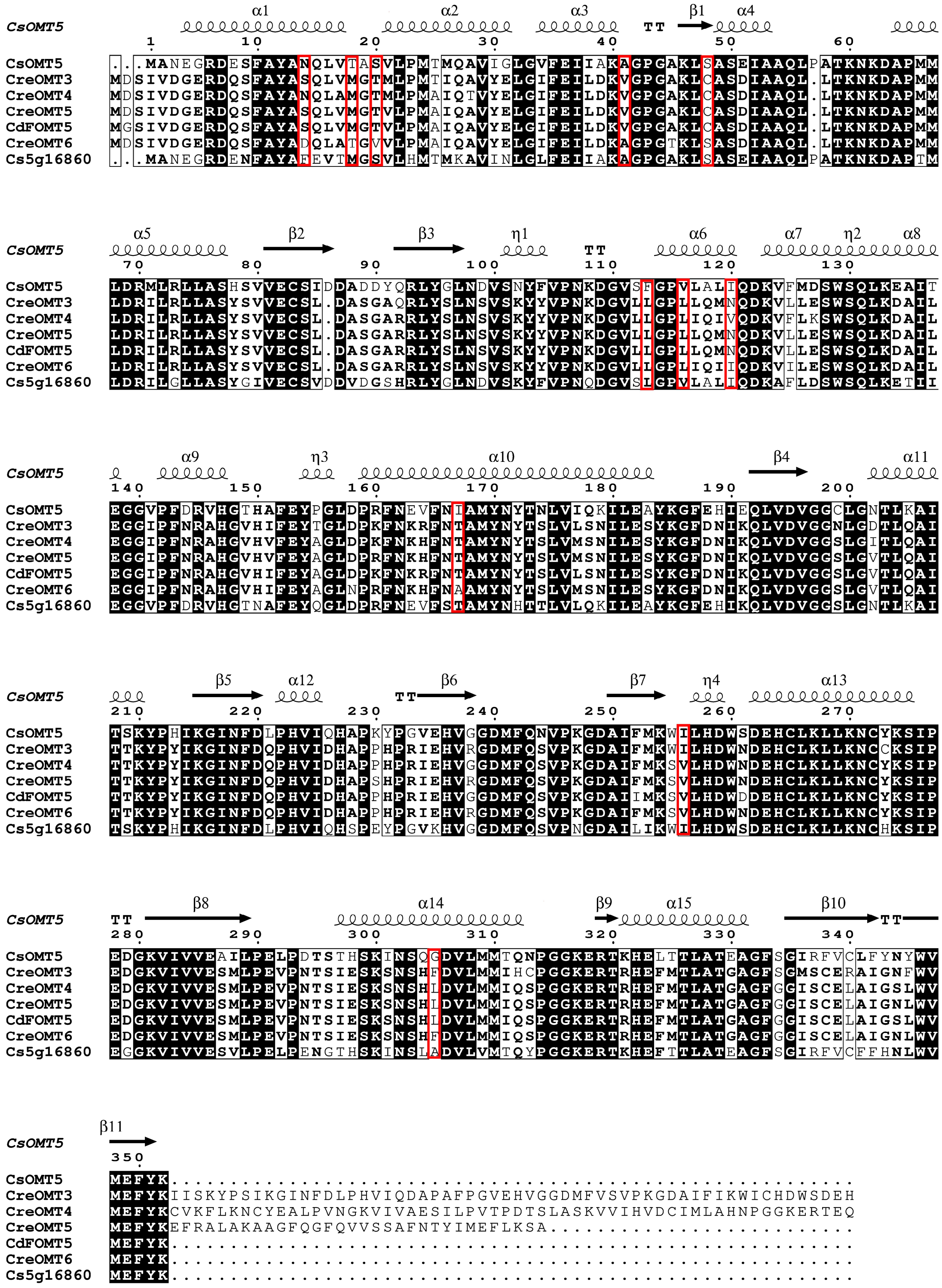

Supplemental Figure 7. Multiple sequence alignment of CsOMT5 with homologous COMTs. Residues targeted for site-directed mutagenesis in this study (N14S, T18M, S20T, A41V, S48C, F113L, V116L, I120N, I120V, I167T, I256V, G305L, G305F) are highlighted in red boxes.

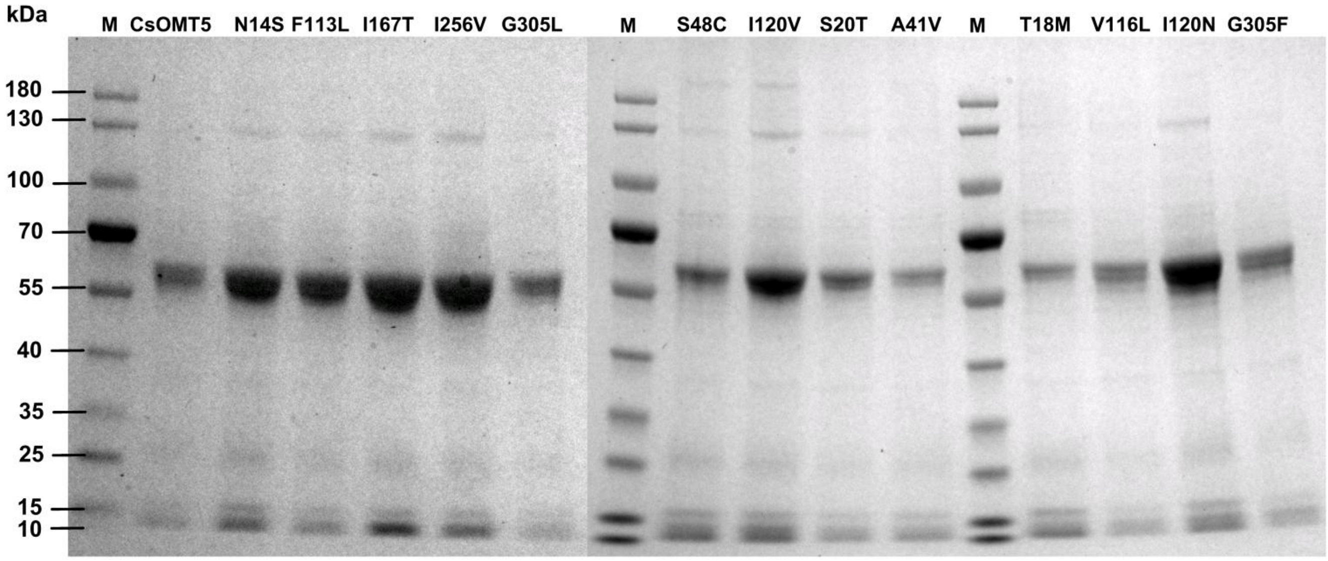

Supplemental Figure 8. SDS-PAGE analysis of recombinant CsOMT5 mutants in *E. coli*.

M, Protein marker.

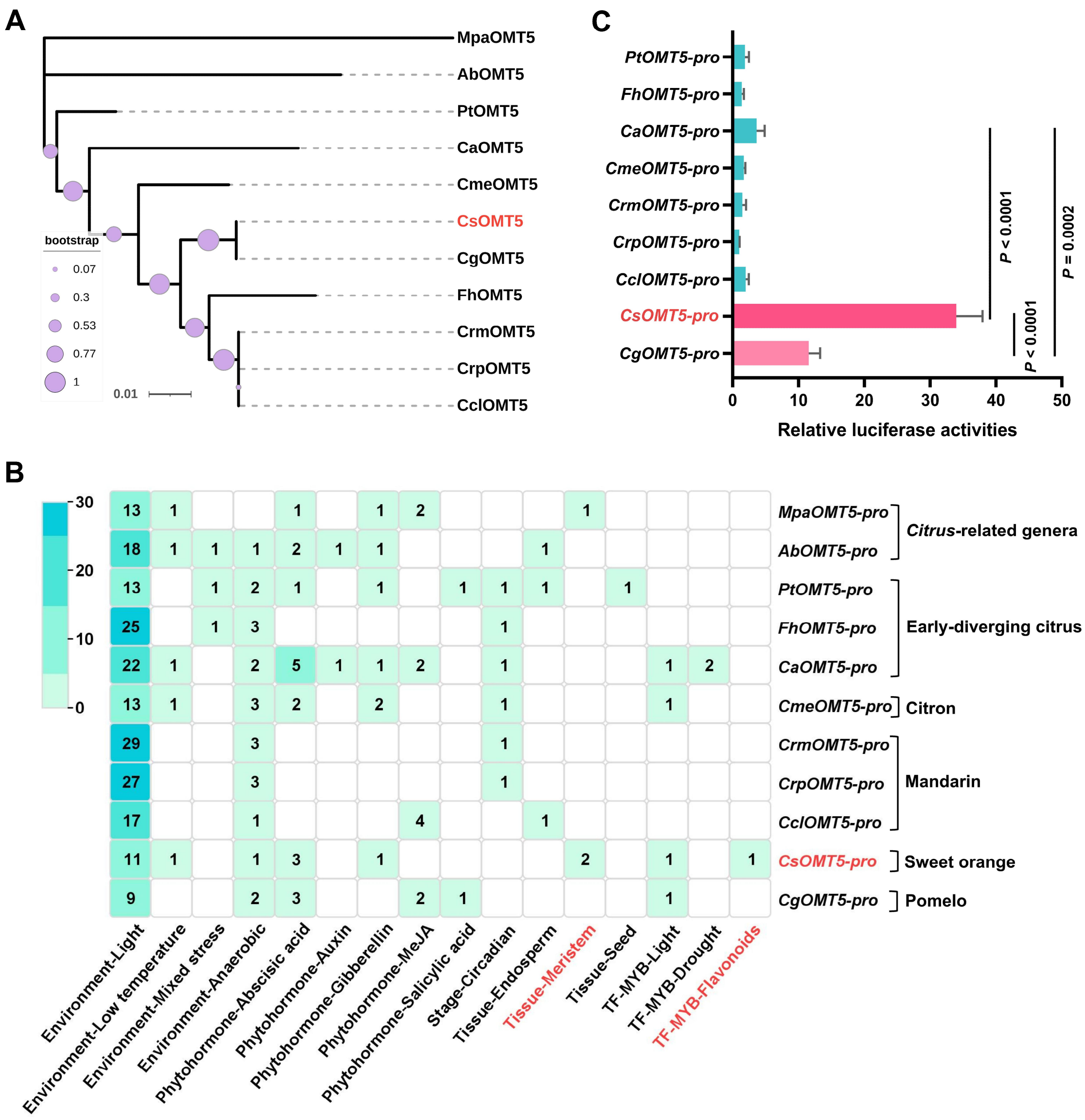

Supplemental Figure 9. Comparative phylogenetic and promoter analysis of CsOMT5 and its homologs in diverse cultivars.

(A) Phylogenetic analysis of CsOMT5 and its homologs in representative cultivars: MpaOMT5 (*M.paniculata*), AbOMT5 (*A.buxfoliata*), PtOMT5 (*P.trifoliata*), FhOMT5 (*F.hindsii*), CaOMT5 (*C.australasica*), CmeOMT5 (*C.medica*), CrmOMT5 (*C.reticulata* 'Mangshan'), CrpOMT5 (*C.reticulata* 'Ponkan'), CclOMT5 (*C.clementina*), CsOMT5 (*C.sinensis*), CgOMT5 (*C.grandis* 'Majiayou'). **(B)** Promoter *cis*-element analysis of *CsOMT5* and its homologous genes in diverse cultivars. *Cis*-elements identified via PlantCARE (https://bioinformatics.psb.ugent.be/webtools/plantcare/html/Menu.html). **(C)** Dual-luciferase assays of *CsOMT5* and its homologous genes in representative citrus cultivars. Data are mean ± SD (n = 3). Statistical significance was assessed by one-way ANOVA followed by Tukey's multiple range test. Dual-luciferase assays were performed using the split-leaf method, following the protocol described by Peng *et al.* [1].

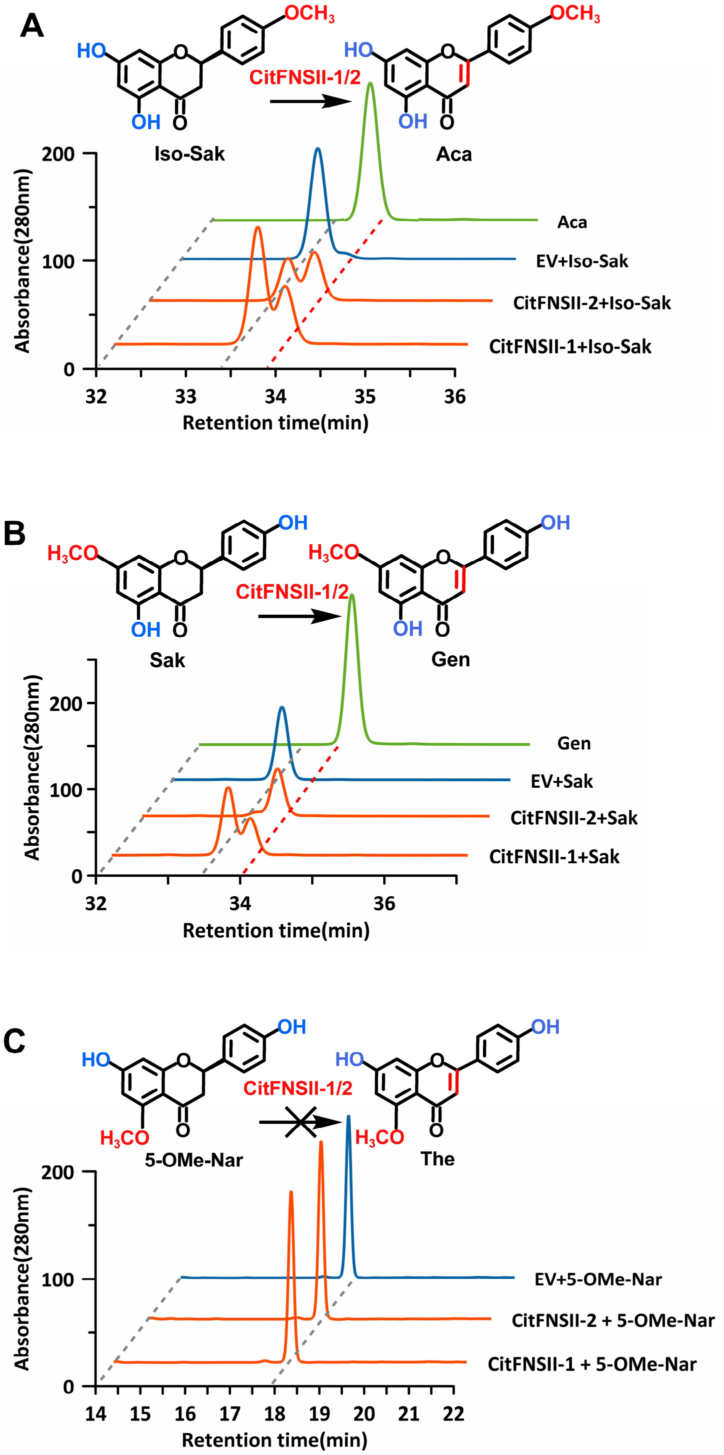

Supplemental Figure 10. *In vivo* yeast enzymatic activity assay between CitFNSII-1/2 and monomethyl ether derivatives of naringenin.

Reaction components are indicated as follows: orange, substrates catalyzed by recombinant CitFNSII-1/2; blue, substrates incubated with the empty vector; green, authentic methylated standards. **(A)** Assay with Iso-Sak (isosakuranetin): Aca, acacetin. **(B)** Assay with Sak (sakuranetin): Gen, genkwanin. **(C)** Assay with 5-OMe-Nar (5-methylnaringenin): The, thevetiaflavone. Yeast expression and enzymatic assays of CitFNSII-1/2 were performed according to our established protocol [2].

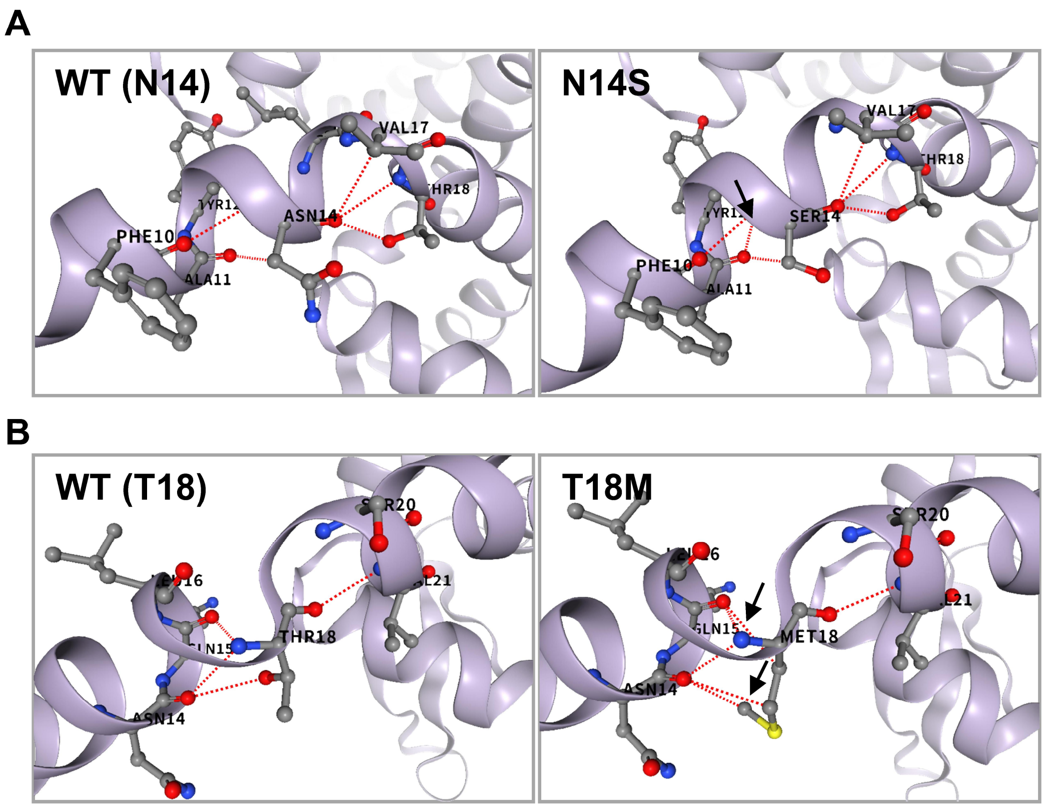

Supplemental Figure 11. Structural analysis of N14 and T18 mutations via DynaMut.

(A) Structural comparison between the wild-type (WT, N14) and the N14S mutant. The predicted stability change ($\Delta\Delta G_{\text{Stability}}$) is 0.05 kcal/mol (stabilizing). **(B)** Structural comparison between the wild-type (WT, T18) and the T18M mutant. The predicted stability change ($\Delta\Delta G_{\text{Stability}}$) is 0.28 kcal/mol (stabilizing). Throughout the figure, red dashed lines represent the local hydrogen bond network, and black arrows indicate newly formed hydrogen bond resulting from mutation.
