# Supplementary material for: A novel C5-O-methyltransferase for naringenin refines the biosynthetic strategy for polymethoxyflavones
Source: Hortic Res. 2026 Apr 6;13(8):uhag128. doi: 10.1093/hr/uhag128 (PMC13411268; doi:10.1093/hr/uhag128)
Supplement: Web_Material_uhag128 [file web_material_uhag128.zip › Supplementary Tables in Word.docx]

| **Supplemental Table 1. Proteins from other organisms used in phylogenetic analysis.** | | |
| --- | --- | --- |
| Protein Name | Organism | UniProt entry |
| AtOMT1 | *Arabidopsis thaliana* (Mouse-ear cress) | Q9FK25 |
| CrCOMT1 (CaroCOMT1) | *Catharanthus roseus* (*Madagascar periwinkle*) (Vinca rosea) | Q8W013 |
| CrOMT2 (CaroOMT2) |  | Q8GSN1 |
| CrOMT6(CaroOMT6) |  | Q6VCW3 |
| CaOMT1 | *Chrysosplenium americanum* (Golden saxifrage) | P59049 |
| CaOMT2 |  | Q42653 |
| CaF3'OMT |  | Q42654 |
| HvOMT1 | *Hordeum vulgare* (Barley) | Q43771 |
| Hv7OMT | *Hordeum vulgare* subsp. *vulgare* (Domesticated barley) | A5YTR4 |
| MsCOMT1 | *Medicago sativa* (Alfalfa) | P28002 |
| MpOMT1A | *Mentha piperita* (Peppermint) (*Mentha aquatica* x *Mentha spicata*) | Q6VMW2 |
| MpOMT1B |  | Q6VMW1 |
| MpOMT2 |  | Q6VMW0 |
| MpOMT3 |  | Q6VMV9 |
| MpOMT4 |  | Q6VMV8 |
| ObFOMT1 | *Ocimum basilicum* (Sweet basil) | K0I977 |
| ObFOMT2 |  | K0II72 |
| ObFOMT3 |  | K0I7Q2 |
| ObFOMT4 |  | K0I210 |
| ObFOMT5 |  | K0ICR0 |
| ObFOMT6 |  | K0I986 |
| ObF8OMT-1 |  | S5DWK8 |
| OsOMT1 | *Oryza sativa* subsp. *japonica* (Rice) | Q6ZD89 |
| OsNOMT |  | Q0IP69 |
| PaF4'OMT | *Plagiochasma appendiculatum* | A0A1X9Y2Y6 |
| ShMOMT1 | *Solanum habrochaites* (Wild tomato) (*Lycopersicon hirsutum*) | F2YTN4 |
| ShMOMT2 |  | F2YTN5 |
| ShMOMT3 |  | M9Z1G5 |
| SlMOMT4 | *Solanum lycopersicum* (Tomato) (*Lycopersicon esculentum*) | A0A088MF62 |
| TaOMT1 | *Triticum aestivum* (Wheat) | Q84N28 |
| TaOMT2 |  | Q38J50 |
| ZmOMT1 | *Zea mays* (Maize) | Q6VWG3 |
| CsOMT21(CasaOMT21) | *Cannabis sativa* (Hemp) (Marijuana) | A0A7J6HVP6 |
| GmOMT1 | *Glycine max* (Soybean) (Glycine hispida) | C6TDI4 |
| GmOMT2 |  | I1NID6 |
| GmOMT3 |  | I1KWG9 |
| GmOMT5 |  | C6TEX3 |
| GmOMT6 |  | [C6TIJ7](https://www.uniprot.org/uniprotkb/C6TIJ7/entry) |
| GmOMT8 |  | A0A0R0GPT0 |
| GmOMT9 |  | I1LC13 |
| GmOMT10 |  | I1JJX3 |
| GmOMT11 |  | A0A0R4J3J0 |
| GmOMT12 |  | A0A0R4J3I7 |
| GmOMT14 |  | A0A0R4J3I5 |
| GmOMT15 |  | K7M862 |
| GmOMT16 |  | K7MUZ8 |
| GmOMT17 |  | I1NID5 |
| GmOMT18 |  | I1KB23 |

| **Supplemental Table 2. Primers used in this study.** | | |
| --- | --- | --- |
| **Experiment purpose** | **Primer name** | **Primer sequence (5′→3′)** |
| E. coli protein expression | Cs1g03800-pET32a-FP | TATCGGATCCGAATTCATGCTGATCATGGAGCTTGCC |
|  | Cs1g03800-pET32a-RP | GGTGGTGGTGCTCGAGTTTGTAGAGTTCAACAAGCCAACAG |
|  | Cs1g12670-pET32a-FP | TATCGGATCCGAATTCATGGCTGCGAATCAAGAAGGG |
|  | Cs1g12670-pET32a-RP | GGTGGTGGTGCTCGAGACTAATGCGACGGCATAATGTAACC |
|  | Cs5g16860-pET32a-FP | TATCGGATCCGAATTCATGGCTAATGAAGGGAGAGACG |
|  | Cs5g16860-pET32a-RP | GGTGGTGGTGCTCGAGCTTGTAGAACTCCATAACCCATAAA |
|  | CsOMT5-pET32a-FP | TATCGGATCCGAATTCATGGCTAATGAAGGGAGAGACG |
|  | CsOMT5-pET32a-RP | GGTGGTGGTGCTCGAGCTTGTAGAACTCCATAACCCAAT |
|  | Cs5g24990-pET32a-FP | TATCGGATCCGAATTCATGGCTTCTATAGCTGATCAGGA |
|  | Cs5g24990-pET32a-RP | GGTGGTGGTGCTCGAGTTTCAAGAACTCCATAACATATAAA |
| RT-qPCR | CsOMT5-qPCR-FP | CTTCCAGAACTGCCTGACAC |
|  | CsOMT5-qPCR-RP | GTTCGCTCCTTTCCCCCTG |
|  | Citrus β-actin | CATCCCTCAGCACCTTCC |
|  | Citrus β-actin | CCAACCTTAGCACTTCTCC |
| Overexpression in citrus peels | CsOMT5-pBI121-FP | GGACTCTAGAGGATCCATGGCTAATGAAGGGAGAGACG |
|  | CsOMT5-pBI121-RP | GACCACCCGGGGATCCCTTGTAGAACTCCATAACCCAAT |
| VIGS in citrus seedlings | CsOMT5-TRV2-FP | GCCTCCATGGGGATCCAATCAATTGGTGACGGCTTCAGTG |
|  | CsOMT5-TRV2-RP | ATGCCCGGGCCTCGAGGTCTCTGATAATCATCAGCATCATC |
| Subcellular localization | CsOMT5-eGFP-FP | CGGTACCCGGGGATCCATGGCTAATGAAGGGAGAGACG |
|  | CsOMT5-eGFP-RP | CGACTCTAGAGGATCCCTTGTAGAACTCCATAACCCAAT |
| Site-directed mutagenesis of CsOMT5 | N14S-FP | ATATGCCagcCAATTGGTGACGGCTTCAGTGC |
|  | N14S-RP | CCAATTGgctGGCATATGCAAAGCTTTCGTCT |
|  | T18M-FP | ATTGGTGatgGCTTCAGTGCTTCCCATGACCA |
|  | T18M-RP | CTGAAGCcatCACCAATTGATTGGCATATGCA |
|  | S20T-FP | TaccGTGCTTCCCATGACCATGCAAGCAGTAA |
|  | S20T-RP | TCATGGGAAGCACggtAGCCGTCACCAATTGATTGG |
|  | A41V-FP | TAGCCAAAgtgGGTCCTGGAGCAAAGCTCTCA |
|  | A41V-RP | AGGACCcacTTTGGCTATGATCTCAAAAACTCCC |
|  | S48C-FP | AAAGCTCtgcGCTTCAGAGATTGCCGCTCAGT |
|  | S48C-RP | CTGAAGCgcaGAGCTTTGCTCCAGGACCAGCT |
|  | F113L-FP | AGATGGTGTCTCActgGGCCCTGTGTTGGCCTTAA |
|  | F113L-RP | CcagTGAGACACCATCTTTATTAGGCACAAAG |
|  | V116L-FP | CCCTctgTTGGCCTTAATTCAGGACAAGGTCT |
|  | V116L-RP | TTAAGGCCAAcagAGGGCCGAATGAGACACCA |
|  | I120N-FP | GGCCTTAaacCAGGACAAGGTCTTTATGGACAGC |
|  | I120N-RP | TGTCCTGgttTAAGGCCAACACAGGGCCGAAT |
|  | I120V-FP | GCCTTAgtgCAGGACAAGGTCTTTATGGACAGC |
|  | I120V-RP | TTGTCCTGcacTAAGGCCAACACAGGGCCGAA |
|  | I167T-FP | TTTCAACaccGCGATGTACAACTATACCAATTTGG |
|  | I167T-RP | ACATCGCggtGTTGAAAACTTCATTGAACCTGGG |
|  | I256V-FP | ATGAAGTGGgtgCTCCACGATTGGAGCGATGA |
|  | I256V-RP | TGGAGcacCCACTTCATAAAAATGGCATCACC |
| Dual-LUC assay | PtOMT5-pro-FP | GCAGCCCGGGGGATCCAAAAAGGAAGAAAATCGGGCTG |
|  | PtOMT5-pro-RP | TTGGCGTCTTCCATGGATTCTCTTGAATTTCCTTATCTGTT |
|  | FhOMT5-pro-FP | GCAGCCCGGGGGATCCTTGTTAATAATAATAATAATTAACG |
|  | FhOMT5-pro-RP | TTGGCGTCTTCCATGGATTCTCTTGAATTTCCTTATC |
|  | CaOMT5-pro-FP | GCAGCCCGGGGGATCCATAAGTATGAAAAAATTAATAATTT |
|  | CaOMT5-pro-RP | TTGGCGTCTTCCATGGATTCTCTTGAATTTCTTACC |
|  | CmeOMT5-pro-FP | GCAGCCCGGGGGATCCCTTCATCTGTTGTCTGCCACA |
|  | CmeOMT5-pro-RP | TTGGCGTCTTCCATGGATTCTCTTGAATTTCTTACCTGTTT |
|  | CrmOMT5-pro-FP | GCAGCCCGGGGGATCCTGTTAATAATAATAATAATTAACGA |
|  | CrmOMT5-pro-RP | TTGGCGTCTTCCATGGATTCTCTTGAATTTCCTTATCT |
|  | CrpOMT5-pro-FP | GCAGCCCGGGGGATCCTGTTAATAATAATAATAATTAACGA |
|  | CrpOMT5-pro-RP | TTGGCGTCTTCCATGGATTCTCTTGAATTTCCTTATCT |
|  | CclOMT5-pro-FP | GCAGCCCGGGGGATCCAATCCCAGGGCTGAACTC |
|  | CclOMT5-pro-RP | TTGGCGTCTTCCATGGATTCTCTTGAATTTCCTTATCTGTT |
|  | CsOMT5-pro-FP | GCAGCCCGGGGGATCCATATCAAAGTAGTCACAAAATGGC |
|  | CsOMT5-pro-RP | TTGGCGTCTTCCATGGATTCTCTTGAATTTCCTTATCTG |
|  | CgOMT5-pro-FP | GCAGCCCGGGGGATCCTTTATCTCTGGAACAAATCTAACCT |
|  | CgOMT5-pro-RP | TTGGCGTCTTCCATGGATTCTCTTGAATTTCCTTATCTGTT |

Underlined sequences represent restriction enzyme site
